# Assessing indoor positioning system: A q-spherical fuzzy rough TOPSIS analysis

**DOI:** 10.1016/j.heliyon.2024.e31018

**Published:** 2024-05-10

**Authors:** Ahmad Bin Azim, Asad Ali, Abdul Samad Khan, Fuad A. Awwad, Emad A.A. Ismail, Sumbal Ali

**Affiliations:** a Department of Mathematics and Statistics, Hazara University Mansehra 21300, Khyber Pakhtunkhwa, Pakistan; b Research Center for Computational Science, School of Mathematics and Statistics, Northwestern Polytechnical University, Xi'an, 710129, China; c Department of Quantitative analysis, College of Business Administration, King Saud University, P.O. Box 71115, Riyadh, 11587, Saudi Arabia

**Keywords:** q-Spherical fuzzy rough sets, Aggregation operators, TOPSIS method, Decision-making to select appropriate technology, Multiple-criteria decision-making

## Abstract

This study investigates advanced data collection methodologies and their implications for understanding employee and customer behavior within specific locations. Employing a comprehensive multi-criteria decision-making framework, we evaluate various technologies based on four distinct criteria and four technological alternatives. To identify the most effective technological solution, we employ the q-spherical fuzzy rough TOPSIS method, integrating three key parameters: lower set approximation, upper set approximation, and parameter q (where q ≥ 1). Our novel approach combines the TOPSIS method with q-spherical fuzzy rough set theory, providing deeper insights into data-driven decision-making processes in corporate environments. By comparing our proposed framework with existing multi-criteria decision-making methodologies, we demonstrate its strength and competitiveness. This research contributes to enhancing decision-making capabilities in corporate settings and beyond.

## 1 Introduction

When it comes to gathering the information needed to investigate the behaviors of customers, one may choose from several different approaches. When compared to other common means of data collection, such as interviews, document reviews, and observations, the questionnaire method is not the one that is used the most. Instead, the most common method is the interview. Research conducted by Kirchberg and Trondle [1] in 2012 includes the use of interviews as a method for analyzing how the experiences of visitors may be compared to and differentiated from those of one another. Utilizing older methods has several problems, one of which is that these methods do not collect the real-time that activities take place. As a direct result of this, the data that was analyzed does not correspond to the actual results in any way. The mentioned literature also provides studies that give the technological basis that is essential to obtaining location data. For example, Fernandez-Liatas et al. [2] 2015 investigated how radio frequency identification (RFID) technology is used in a variety of healthcare processes. Yuanfang et al. [3] 2016 were able to determine the locations of users who were inside a restricted space by using signals that were sent to users' mobile phones from Wi-Fi. These signals were delivered to users' phones when the users were within a limited area. ZigBee technology was used to gather data, which Huang, and Chan [4] in 2011 then used to determine which item was situated closer to a certain area than the others. Certain establishments sell items to the public that use cameras to monitor the actions of their customers at different times throughout the day. Wu et al. [5] 2015 used footage captured by cameras installed within the stores to get insight into customer habits and movements. Dogan et al. [[6], [7], [8], [9]], defined different methodologies for the indoor customer path using fuzzy decision-making, process mining, intuitionistic fuzzy clustering, and Levenshtein-based fuzzy kNN. Approaches such as spherical fuzzy TOPSIS [10], spherical fuzzy VIKOR [11], spherical fuzzy WASPAS [12], spherical fuzzy AHP [13], and spherical fuzzy QFD [14] are some examples of recent developments in this area. The marketing process, its tools, and its function have all transformed as a direct consequence of the growth of mobile technology. According to Celikkan et al. [15], in 2011, it is very necessary to understand the requirements and preferences of the clients to deliver effective communication. In addition to this, tailored marketing messages and suggestions have become highly well-liked, and buyers even prefer to receive these messages while they are shopping in shops Merad et al. [16], in 2016.Using several technologies to keep track of customers when they are physically present at a store has made it possible to conquer the barrier of successfully combining online and offline distribution channels. It is possible to utilize behavioral analytics to analyze user requests from internal locations to provide the most effective messaging to customers. This may be accomplished by collecting data from users. Oosterlinck et al. [17] in 2017). Information on the areas that are frequented by customers is included in the data that is created by behavioral analytics systems. For instance, personalized offers such as gift-giving vouchers for purchases during the most time-consuming department of a customer who has spent more than 90 min in the shop, assuming the customer is hungry, not only enhance sales but also increase customer loyalty. This is because the consumer is more likely to feel appreciated and valued by the business. Customers who have been in the shop for more than 1 h and 90 min are eligible to get deals of this kind. The results of the study carried out by Merad et al. [16], in 2016 indicate that seven out of ten transactions are finalized as a direct consequence of a decision made while in the store. This highlights how important it is to know the requirements of the clients and provide individualized advice. When compared to collecting data from customers at physical locations, collecting data from customers via websites or mobile apps is a relatively straightforward process. To operate an online business effectively, it's crucial to employ specific technologies and software solutions like RFID (Radio Frequency Identification), Wi-Fi (Wireless Fidelity), and Bluetooth for precise customer data collection. Although a great variety of techniques for the collection of data at retail locations have been developed, there is widespread consensus that Yucel et al. [18], in 2012. Every strategy comes with its own set of advantages, in addition to certain limitations. The process of selecting the technology that is best suited for carrying out behavioral analytics on the premises is an example of a circumstance that calls for the use of multiple criteria decision-making (MCDM). When there are several different alternatives to choose from and criteria to consider, this sort of difficulty develops. The origins of fuzzy set theory [19] may be traced back to the standard crisp set theory. In addition, Zadeh introduced the notion of a fuzzy set, which focuses solely on the grades that received a positive evaluation. Atanassov [20] introduced the intuitionistic fuzzy set (IFS) to expand upon the concept of fuzzy sets. The intuitionistic fuzzy set (IFS) incorporates both the positive and negative grades with the stipulation that their cumulative value should not exceed 1. For instance, IFS accommodates assessments of both favorable and unfavorable academic performance. Over time, the significance of IFS has increased since its initial introduction, leading to widespread adoption in decision-making and problem-solving contexts. In 2014, Coung and Kreinovich expanded upon the concepts of fuzzy sets and IFS introduced a groundbreaking notion known as the picture fuzzy set (PFS) [21]. This innovation provided a fresh perspective within this field of study. Within the framework of PFS, the author delved into the categorization of grades into positive, neutral, and negative classifications. In a 2015 publication, Zhang et al. [22] proposed an approach to enhance decision-making processes by incorporating diverse attribute groups. Their method involved the use of intuitionistic fuzzy Frank power aggregation operations. Seikh and Mandal [23] suggested the development of fuzzy Dombi aggregation operators, designed for simplicity and employed in the context of addressing challenges in multiple criteria decision-making (MCDM). Moreover, Zeng et al. [24] put forward an original approach for multicriteria decision-making. It's crucial to consider that within the context of an intuitionistic fuzzy set (IFS), a constraint is placed on the sum of functions (ζ+η)∈[0,1]. To address this concern, Yager [25] introduced the notion of Pythagorean fuzzy set, denoted by PyFS, serving as an extension of the IFS. To establish this mentioned extension, it's necessary to analyze the prerequisite condition, which is expressed as $0\leq {\left(\zeta \right)}^{2}+{\left(\eta \right)}^{2}\leq 1\text{.}$ PyFS stands as a mathematically sound tool that imparts enhanced flexibility to the decision-maker in the realm of fuzzy set theory, enabling more adaptable utilization of existing information. After the publication of PyFS, various researchers attempted to develop their iterations by integrating supplementary aggregation operators (AOs). Consequently, Akram et al. [26] introduced specific fuzzy Pythagorean Dombi aggregation operators. Furthermore, Garg [27] introduces a range of PyF aggregating processes that are contingent upon the degree of confidence and implements them within the context of data mining situations. Furthermore, Wang and Garg [28] introduced a computational procedure for multiple attribute decision-making (MADM) that leverages the Pythagorean fuzzy set (PyFS) concept and integrates interactive Archimedean norm operations. The research undertaken by Wu et al. [29] effectively demonstrated the adaptability of the MAGDM approach through the application of an information fusion method in conjunction with uncertain Pythagorean fuzzy sets. The concept of PyFS has attracted substantial scholarly interest due to its promising utility within grading systems. However, it's important to emphasise a circumstance in which a positive grade (PG) of 0.8 and a negative grade (NG) of 0.7 are assigned, and the sum (0.8^2^+0.7^2^) surpasses the range of [0,1]. Therefore, it may be concluded that PyFS is not appropriate for addressing this specific issue in its present form. The notion of a q-rung orthopair fuzzy set (q-ROFS) was suggested by Yager [30] as an extension of intuitionistic sets (IFS) and Pythagorean fuzzy sets (PyFS) to facilitate this process. In a recent academic study, Xing et al. [31] formulated a range of point-weighted aggregation operators (AOs) tailed for q-ROFS, delving into their potential utilization within contexts of multiple criteria decision-making (MCDM). Additionally, Liu and Wang [32] have put forward numerous aggregation operators grounded in multiple q-ROFSs, primarily targeting resolving challenges in multiple attribute decision-making scenarios. Aggregation operators (AOs) have been established for these purposes. It is worth noting that the conceptual frameworks only refer to the grades PG and NG. The importance of the abstinence grade (AG) is often disregarded, despite its applicability in many real-world situations. The issue was first recognized by Cuong and Kreinovich [33], who established the notion of a picture fuzzy set (PFS) in their published literature. The use of PFS is a more efficacious approach to tackling the inherent ambiguity and uncertainty that are often encountered in Multi-Criteria Decision Making (MCDM) situations. It's significant to highlight that the PFS follows the fundamental guideline that the combined values of (ζ,η,ξ)∈[0,1] are limited within the range of 0 and 1. In their study, Wei [34] examines a range of PF Hamacher aggregation operators (AOs) that are grounded on the theory of possibility fuzzy sets (PFS). If all the membership grades for PFS are exclusively 0.5 (positive), 0.4 (negative), or 0.2 (neutral), it may be inferred that PFS fails to fulfill its primary criterion, therefore rendering it ineligible to be regarded as genuine formation. The reason for this discrepancy is that the sum of 0.5, 0.2, and 0.4 does not lie inside the interval [0, 1] in the given context. Gündoğdu and Kahraman [10] suggest the use of a spherical fuzzy set (SFS) as a promising approach to tackle this problem. In recent years, there has been a growing scholarly interest in the subject of SFS. In recent research, Ullah et al. [35] proposed a multi-attribute decision-making technique that employs complicated T-spherical fuzzy Frank prioritised aggregation operations to improve decision-making processes. Hussain et al. [36] designed complicated spherical fuzzy Aczel Alsina aggregation operators and used them to evaluate electric automobiles. These publications contribute significantly to the design and implementation of fuzzy aggregation operators in decision science. The study by Kakati et al. [37] delves into the analysis and practical implementation of rectified complex T-spherical fuzzy Dombi-Choquet integral operators for diabetic retinopathy detection through fundus images. On the other hand, Sarkar et al. [38] explore Sugeno-Weber triangular norm-based aggregation operators within the context of T-spherical fuzzy hypersoft, aiming to provide novel insights and applications in the field of information sciences. The references [37,38] are relevant to the approach of the q-spherical fuzzy rough TOPSIS method. Although their focus is on a different aspect of fuzzy logic, their research contributes to the broader understanding of fuzzy systems and their applicability in complex decision-making contexts. This complements the q-spherical fuzzy rough TOPSIS method by providing additional insights into fuzzy aggregation techniques, which can potentially enhance the robustness and effectiveness of the proposed approach. In their efforts to tackle the issue of uncertainty, Kahraman and his research team (reference [39] introduced the innovative concept of a q-spherical fuzzy set (q-SFS). This innovative concept has proven to be exceptionally valuable in assisting them in making well-informed decisions. It is widely acknowledged as an extension of the conventional "q-spherical fuzzy set." Every characteristic of the q-SFS framework is rated as positive, neutral, or negative. It is important to use the condition $0\leq {\left(\zeta \right)}^{q}+{\left(\eta \right)}^{q}+{\left(\xi \right)}^{q}\leq 1$. The q-spherical fuzzy set includes a wide variety of replies, such as affirmative, negative, unsure, and even abstention from responding. The criterion states that the sum of the q powers of ζ, η, and ξ should not exceed one. q-SFS with the parameter q provides decision makers with a wider range of alternatives, allowing them to define their preferences for membership, non-membership, and ambiguity.

The concept of rough sets (RS) was first introduced by Pawlak [40,41] as a means of dealing with uncertainty. When examined from a mathematical perspective, this configuration demonstrates attributes that could be construed as vagueness and indeterminacy. Rough set theory (RST) is a modification of the traditional set theory, that uses the notion of connection to elucidate the operations of information systems. Researchers have acknowledged that the applicability of the equivalence relation in Pawlak's relational semantic theory is subject to notable constraints in a range of real-world situations, a point emphasized by multiple scholars. Consequently, several researchers have expanded upon Pawlak's rough set theory [42,43]. IFSs, PyFSs, and q-ROFSs, while serving different purposes, are all confined to binary choices, such as positive or negative choices. Nevertheless, it is essential to recognize that individuals' viewpoints are never as straightforward as a binary affirmation or negation. Consider exploring the process of voting as an illustrative instance. There exist four distinct potential outcomes: casting a vote in favor (voting yes), casting a vote against (voting no), refraining from casting a vote (abstaining from voting), or not participating in the voting process altogether. The specific incident in question lacks a valid explanation within the existing recognized framework. It's worth mentioning that both the PFS and the SFS can be employed to address such issues, although they come with their inherent limitations. The utilization of the q-spherical fuzzy set has been identified as the optimal approach for resolving this problem. Additionally, it's crucial to ensure that the existing theories adeptly handle any possible concerns or challenges within their respective frameworks or contexts, as exemplified below. The extent of plagiarism is considerably high. Swift action is imperative to entirely rectify this matter.

The theories of IFRS [44], PyFRS [45], and q-ROFRS [46,47] are well-established in the field and have been acknowledged for their contributions. However, it is essential to acknowledge that these theories encounter substantial limitations when striving to encompass all three potential grades within a given dataset: positive, neutral, and negative grades. The examination of voting can be approached through the utilization of a theoretical framework termed a picture fuzzy rough set (PFRS). Nevertheless, the existence of lower and upper approximations, represented as $\left(\underline{\zeta }+\underline{\eta }+\underline{\xi }\;\right)\in \left[0,1\right]$ and ($\bar{\zeta }+\bar{\eta }+\bar{\xi }\;)\in \left[0,1\right]$, imposes certain constraints. The level of plagiarism is excessively high. Immediate and decisive measures are necessary to completely address this issue. Nevertheless, it is crucial to acknowledge that when decision-makers are presented with information in the format of q-SFRS, involving both the lower and upper approximations like {(0.7,0.8,0.9), (0.9,0.8,0.7)} and so on, it is important to acknowledge that the combine values of the lower and upper approximations exceed the interval [0,1]. This suggests that the values (0⩽̸0.7^2^+0.8^2^+0.9^2^⩽̸1) and (0⩽̸0.9^2^+0.8^2^+0.7^2^⩽̸1) are inappropriate with the SFRS framework, hence, this imparts a limitation on the extent to which the SFRS concept can be effectively applied. The notions of q-SFRS were first presented by Azim et al. [48] in their research paper published in 2023. This fuzzy set combines the advantages inherent in both the RS and the q-SFS. This research introduces a practical approach to decision-making within the framework of q-spherical fuzzy rough sets, thereby expanding the existing knowledge in this field. Within q-SFRS, three distinct parameters involve lower and upper approximations. Our main objective in this study is to advance future research by devising novel aggregation operators alongside defuzzification methods. After a comprehensive analysis, it becomes clear that the concept of q-SFRSs holds substantial potential as an innovative idea, thereby paving the way for numerous opportunities in future research endeavors. The approach referred to as Techniques for Ordered Preference by Similarity to an Ideal Solution (TOPSIS) was first introduced by Hwang and Yoon [49] in the year 1981. The primary objective of this tool is to assist in the identification and evaluation of the optimal option from a restricted range of criteria. The acronym TOPSIS represents Techniques for Ordered Preference by Similarity to the ideal solution. In recent years, many research papers have further developed this approach by integrating fuzzy sets. Interval type-2 fuzzy TOPSIS introduced by Wu et al. [50] offers a robust solution for large-scale group decision-making problems enriched with social network information. Onu et al. [51] presented a comprehensive evaluation framework using a fuzzy TOPSIS multi-criteria decision analysis model to assess sustainable acid rain control options. Ostaysi [52] introduces a decision model integrating AHP with TOPSIS-Grey for selecting information technology, with a focus on content management systems. Estrella et al. [53] propose a hesitant linguistic fuzzy TOPSIS model tailored for heterogeneous contexts, particularly in selecting firms within university techno parks. Onar et al. [54] demonstrate the multicriteria evaluation of cloud service providers utilizing Pythagorean fuzzy TOPSIS, providing insights into efficient decision-making processes in cloud service selection. Gündoğdu and Kahraman [55] presented a novel methodology for the fuzzy Technique for Order of Preference by Similarity to the Ideal Solution (TOPSIS) technique, including the use of growing interval-valued spherical fuzzy sets. q-spherical fuzzy rough TOPSIS integrates the concepts of rough set theory and q-spherical fuzzy sets into the Technique for Order of Preference by Similarity to the Ideal Solution (TOPSIS) framework. q-spherical fuzzy TOPSIS, on the other hand, focuses solely on the integration of q-spherical fuzzy sets with the TOPSIS methodology without incorporating rough set theory. The primary distinction lies in the incorporation of rough set theory in q-Spherical Fuzzy Rough TOPSIS, which adds a layer of uncertainty handling compared to q-spherical fuzzy TOPSIS. Due to the inclusion of rough set theory, q-spherical fuzzy rough TOPSIS may be more computationally complex than q-spherical fuzzy TOPSIS, which relies solely on q-spherical fuzzy sets. While both methods aim to enhance decision-making in uncertain environments, the choice between them depends on the specific requirements of the decision problem and the level of complexity that decision-makers are willing to accommodate. understanding the difference between q-SFR TOPSIS and q-SF TOPSIS is crucial for researchers and practitioners seeking to apply fuzzy decision-making methodologies effectively in various real-world scenarios. In the year 2019, KutluGündoğdu and Kahraman [56] introduced a novel VIKOR technique that incorporates the use of spherical fuzzy sets. The researchers used this methodology in the context of warehouse location selection. In the domain of Industry 4.0, Azim et al. [57] introduced a method for project prioritization using the q-SFR analytic hierarchy process in 2023. Likewise in 2023, Ali et al. [58] initially proposed the concept of averaging aggregation operators within the framework of ${q-ROPFS}_{t}S$. Moreover, the researchers explored the potential applications of these operators in scenarios involving multiple attribute decision-making (MADM).

### 1.1 Motivation

The motivation behind this article stems from recognizing the greater flexibility offered by q-SFRS compared to traditional sets like possibility fuzzy sets (PFS) and spherical fuzzy sets (SFS) in addressing decision-making (DM) problems. It addresses the complexities of multi-attribute decision-making (MADM) problems influenced by imprecise factors within the q-SFRS environment. By highlighting the limitations of existing operators, the article proposes a state-of-the-art method to overcome these challenges, yielding significant findings across various information categories represented by q-SFRS data. Building upon the simplicity and comprehensiveness of the Technique for Order Preference by Similarity to Ideal Solution (TOPSIS), the article introduces the concept of (TOPSIS) within the context of q-SFRS to address challenging decision-making problems. This novel approach aims to ensure more accurate and precise results in real-life MADM situations. Pioneered by Azim et al. [48], the enriched q-SFRS framework demonstrates broader applicability and adaptability to diverse circumstances, paving the way for further research and development in decision science. The integration of q-SFRSs through the TOPSIS method presents intriguing potential for analysis in dynamic decision-making fields, enhancing flexibility and precision across various domains. The utilization of q-spherical fuzzy rough TOPSIS to address uncertainty and hesitancy in real-world decision situations has gained popularity, leveraging the flexible background offered by q-SFRS theory to interpret vague information effectively. Meanwhile, the well-established TOPSIS method ranks alternatives based on their proximity to the ideal solution and distance from the negative solution, making it a robust multi-criteria decision analysis approach. Integrating q-SFRSs with TOPSIS aims to overcome the limitations of conventional decision-making methods when handling rough and vague data. This study aims to advance decision knowledge by proposing a novel and practical approach for decision-makers to navigate complex decision scenarios. By amalgamating TOPSIS with q-SFRSs, the research endeavors to equip decision-makers with a sophisticated and accurate decision-support method, particularly in areas characterized by imprecision, hesitation, and complexity. Recognizing the shortcomings of standard decision models, especially in dealing with imprecise and random data, the rationale for exploring novel techniques such as merging TOPSIS with q-spherical fuzzy rough sets is emphasized. This research strives to provide a comprehensive and adaptable decision validation framework, fostering the advancement of decision knowledge in real-world applications.

The purpose of writing this article is to explain the motivation behind its creation.

1. In terms of studying DM problems, q-SFRS offers greater flexibility compared to PFS and SFS.
2. In a scenario involving q-SFRS, identifying the optimal alternative poses a significant.
3. The challenge is a complex MADM problem influenced by imprecise factors. The current MADM approaches solely rely on picture fuzzy and spherical fuzzy numbers to represent the evaluation data. However, this approach may result in data manipulation, potentially leading to inaccuracies. Consequently, we require a more comprehensive model to further explore alternative possibilities.
4. This article aims to define TOPSIS within the context of q-SFRS, to address challenging decision-making problems.
5. Since TOPSIS is a simple yet innovative method for addressing decision-making problems, this article also acknowledges the groundbreaking nature of TOPSIS.
6. In comparison to existing operators, TOPSIS offers more precise and accurate decision outcomes when applied to real-life MADM situations that rely on the q-SFRS environment. TOPSIS is a simple and concise approach used to evaluate a single option chosen from a list of multiple options.
7. The proposed technique addresses the limitations and drawbacks of existing operators by offering a more comprehensive approach that yields excellent results for various types of data, including q-SFRS information, picture fuzzy, and spherical fuzzy data. This is feasible because the suggested operators have a broader scope.

### 1.2 Research gap

The study aims to address the gap in decision-making methodologies by proposing a novel approach that integrates the q-spherical fuzzy rough sets (q-SFRS) theory with the Technique for Order of Preference by Similarity to the Ideal Solution (TOPSIS) method. The motivation behind this integration stems from the recognition of the limitations of existing decision models, particularly in handling imprecise and uncertain data prevalent in real-world decision scenarios. Despite the availability of theories like picture fuzzy sets (PFS) and spherical fuzzy sets (SFS), they often fail to provide sufficient flexibility to address complex MADM problems influenced by imprecise factors.

The research question arises from the necessity to bridge this gap in decision science: How can we develop a more comprehensive and adaptable decision validation framework to handle the complexities of real-world decision-making scenarios characterized by imprecision, hesitation, and complexity?

To address this question, the study proposes the integration of q-SFRS with TOPSIS, leveraging the flexibility and robustness offered by both theories. By amalgamating these methodologies, the research endeavors to provide decision-makers with a sophisticated and accurate decision support system capable of navigating the intricacies of modern decision environments. The gap lies in the inadequacy of existing decision models to effectively handle the challenges posed by imprecise and uncertain data in real-world decision scenarios. This gap necessitates the development of a more comprehensive and adaptable framework, which the proposed integration of q-SFRS with TOPSIS aims to fulfill. The fusion of these theories offers a promising avenue for advancing decision science and addressing the shortcomings of conventional decision models.

## 2 .Objectives

The fundamental building blocks of every theory are its operational guidelines, which serve as a catalyst and are necessary to form the framework and make the theory work. Without these fundamental principles, a theory could not make sense and be unable to provide useful answers to issues. Thus, to develop reliable and useful theories, understanding and analyzing these operational laws' fundamental attributes is essential. Recognizing the importance of these fundamental operational laws to build reliable and successful theories. Understanding the significance of fundamental operating rules enables us to build more creative and robust frameworks that can handle the problems of the modern world. The idea of q-SFRSs and their use in MADM problems was first presented by Azim et al. [48]. The q-SFRS framework now has a wider variety of applications and more adaptability in various situations thanks to these additional regulations. These generalized operational laws provide new avenues for future research and development by enabling q-SFRS to tackle a greater variety of challenging challenges. The discovery of these laws is a major contribution to science as it offers the foundation for more thorough and innovative approaches to problem-solving in the real world.

Talk about the rationale behind the combination of q-SFRSs and the Technique for Order of Preference by Similarity to Ideal Solution (TOPSIS) offers a fascinating avenue of investigation in the dynamic field of decision-making. The goal of this fusion is to improve the resilience and accuracy of decision-making processes across several domains. Adopting q-spherical fuzzy rough TOPSIS was motivated by its capacity to manage the ambiguity and uncertainty present in real-world decision scenarios. A more realistic portrayal of complicated decision environments is made possible by the flexible framework offered by the q-SFRS theory, which may be used to handle and model imprecise information. As an alternative, TOPSIS is ranked according to how far away an alternative is from the negative solution and how close it is to the ideal answer. TOPSIS is a well-known technique for multi-criteria decision analysis. Our goal with merging q-spherical fuzzy rough sets with TOPSIS is to overcome the drawbacks of conventional decision models in handling rough and uncertain data. By offering decision-makers a fresh and practical method for navigating complex choice environments, this research aims to further the field of decision science. It is expected that the combination of TOPSIS with q-spherical fuzzy rough sets would provide a more sophisticated and accurate decision support system, especially in domains where imprecision, uncertainty, and roughness are common. The realization that accurate and clear information is rarely a defining feature of real-world decision issues serves as the inspiration. This work aims to provide decision-makers with a tool that can handle the complexities and subtleties present in complicated decision-making scenarios by embracing the synergy between q-SFRSs and TOPSIS. The ultimate objective is to provide a strong and flexible framework for decision assistance to aid in the development of decision science.

1. Implementing q-spherical fuzzy rough TOPSIS, highlighting how these sets can manage ambiguity and uncertainty in practical decision-making situations.
2. Describe the flexible framework that the q-SFRS theory offers for handling and modeling imprecise information. Stress how crucial it is to provide a depiction of complicated decision contexts that is more realistic.
3. Recognize the shortcomings of conventional decision models, particularly in the context of imprecise and erratic data. Create the conditions for investigating novel methods such as integrating TOPSIS with q-spherical fuzzy rough sets.

**Fig. 1:**
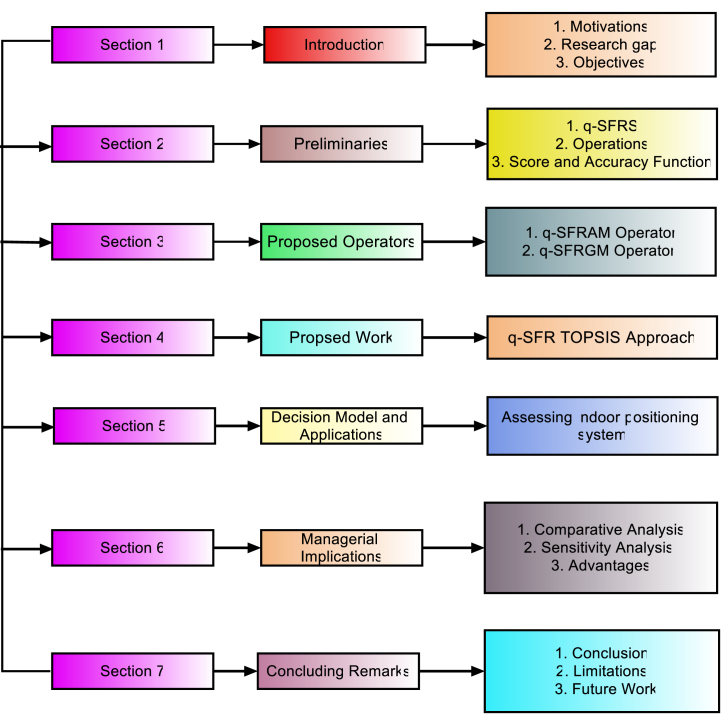
Structure of the research article.

The subsequent sections of the study are structured as follows:

Section 2 provides an all-encompassing overview of a range of concepts, encompassing FS, PFS, SFS, q-SFS, RS, and q-SFRS. This comprehensive introduction shows the stages for the subsequent sections. In section 3, we embark on an in-depth exploration of the operational laws and aggregation operators toiled for the q-SFRNs. For calculations and aggregations within the domain of q-SFR TOPSIS, these fundamental laws and aggregation operators act as the cornerstone. The q-SFR TOPSIS method is explained in detail in section 4. It goes into detail about this method's implementation and practical use, especially when assessing indoor location-tracking technologies. In section 5, we demonstrate how to evaluate indoor location tracking technologies using the q-SFR TOPSIS method. We present the q-spherical fuzzy rough TOPSIS approach as a useful tool for evaluating and contrasting technologies. In section 6, we look at the management implications of the q-SFR TOPSIS techniques. We stress the relevance of sensitivity analysis in determining the robustness of the methods, as well as the significance of comparative analysis and advantages for evaluating multi-criteria decision-making rankings. In this concluding section 7, where we summarize the major findings and limitations and stress the study's overall significance, the research ends. Fig. 1 represents the layout of this research article.

## 3 .Preliminaries

We are prepared to examine a variety of mathematical ideas in this section, starting with a close examination of FS, PFS, SPS, q-SFS, and RS.

Definition 2.1 In 1965, Zadeh [19] proposed the idea of a fuzzy set as an extension of the conventional crisp set. The formal definition of a fuzzy set can be represented mathematically as follows:

(1) $$A=\{\left\langle x,\zeta_{A}\left(x\right)\right\rangle :x∊X\}$$

**Fig. 2:**
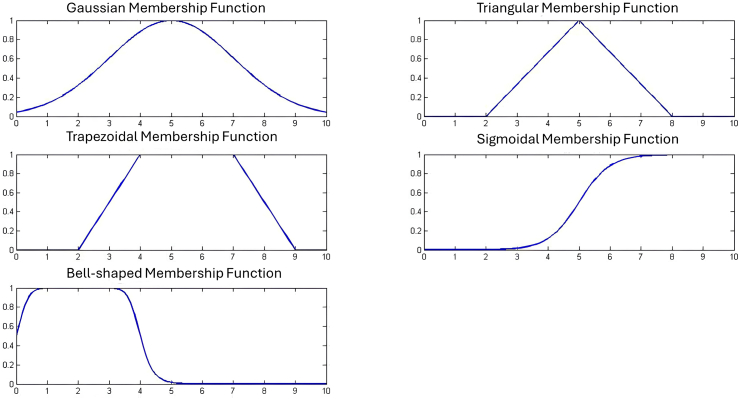
Some graphical representations of fuzzy spaces.

Where $0\leq \zeta_{A}\left(x\right)\leq 1\text{.}$ Fig. 2 represents the graphical representations of fuzzy spaces.

Definition 2.2 In 1986, Atanassov [20] proposed the intuitionistic fuzzy set (IFS) as an extension of the fuzzy set. The formal mathematical representation of an IFS is as follows.

(2) $$A=\{\left\langle x,\zeta_{A}\left(x\right),\xi_{A}\left(x\right)\right\rangle :x∊X\}$$

Where $0\leq \zeta_{A}\left(x\right)+\xi_{A}\left(x\right)\leq 1$.

**Fig. 3:**
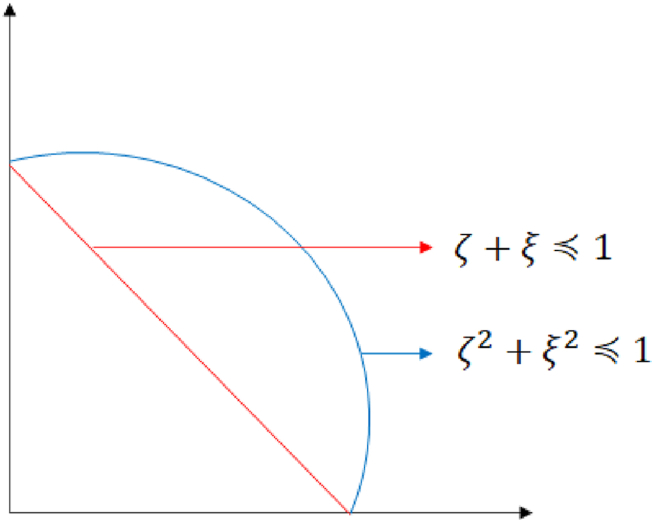
A comparison of the differences between Pythagorean and intuitionistic fuzzy spaces.

Definition 2.3 [25] Let X be a non-empty finite set. A PyFS A over x∊X is defined as follows:

(3) $$A=\{\left\langle x,\zeta_{A}\left(x\right),\xi_{A}\left(x\right)\right\rangle :x∊X\}$$

where $\zeta_{A}\left(x\right)$ and $\xi_{A}\left(x\right)$ represent the MD and NMD of A respectively such that $\xi_{A}\left(x\right),\eta_{A}\left(x\right)$ ∈[0,1] and where $0\leq {\left(\zeta_{A}\left(x\right)\right)}^{2}+{\left(\xi_{A}\right)}^{2}\leq 1$. Fig. 3 represents the comparison between Pythagorean and intuitionistic fuzzy spaces.

Definition 2.4 Building on the fundamental principles of FSs and IFSs, Cuong and Kreinovich [21] introduced the idea of a picture fuzzy set in 2014. Its definition can be expressed mathematically as follows:

(4) $$A=\{\left\langle x,\zeta_{A}\left(x\right),\eta_{A}\left(x\right),\xi_{A}\left(x\right)\right\rangle :x∊X\}$$

Where 0≤ $\zeta_{A}\left(x\right)+\eta_{A}\left(x\right)+\xi_{A}\left(x\right)\leq 1$.

**Fig. 4:**
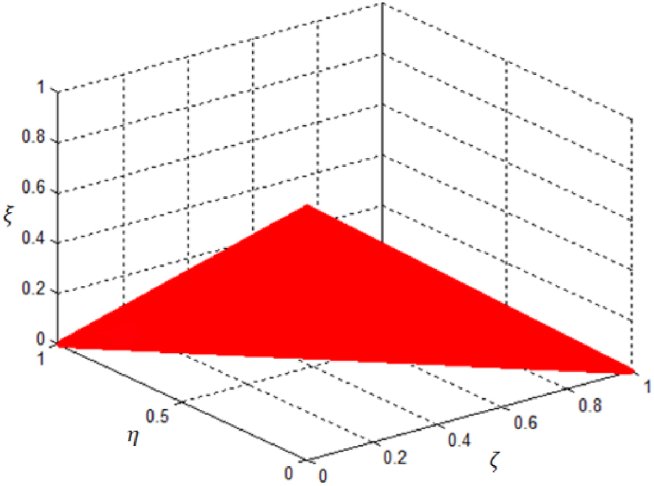
Picture membership grade space.

The following symbols represent the representation of the membership functions for a fuzzy set in this situation, which includes positive, neutral, and negative aspects: $\zeta_{A}\left(x\right)\left(x\right)$: X → [0,1], $\eta_{A}\left(x\right)$: X → [0,1] and $\xi_{A}\left(x\right)$: X → [0,1] respectively. Fig. 4 represents the graphical representations of picture fuzzy space.

Definition 2.5 Gündoğdu et al. [10] introduced the idea of a spherical fuzzy set in 2019, further advancing the picture fuzzy set framework. The concept can be expressed in the following way from a mathematical standpoint:

(5) $$A=\{\left\langle x,\zeta_{A}\left(x\right)\left(x\right),\eta_{A}\left(x\right),\xi_{A}\left(x\right)\right\rangle :x∊X\}$$

Where ${0\leq \left(\zeta_{A}\left(x\right)\right)}^{2}+{\left(\eta_{A}\left(x\right)\right)}^{2}+{\left(\xi_{A}\left(x\right)\right)}^{2}\leq 1$.

**Fig. 5:**
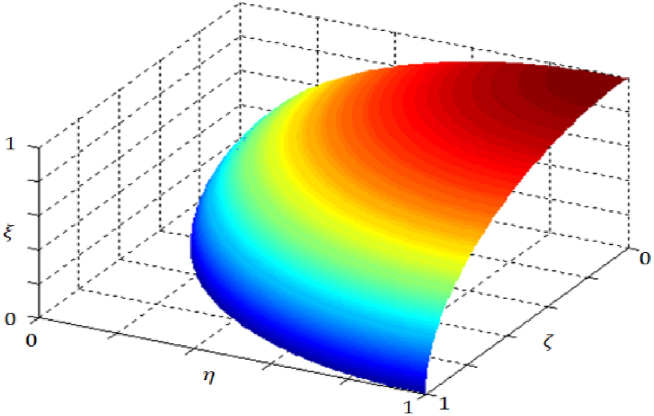
The condition ${0\leq \left(\zeta_{A}\left(x\right)\right)}^{2}+{\left(\eta_{A}\left(x\right)\right)}^{2}+{\left(\xi_{A}\left(x\right)\right)}^{2}\leq 1$ describes a spherical fuzzy set-in three-dimensional space.

Where the positive, neutral, and negative membership function for a fuzzy set is represented by $\zeta_{A}\left(x\right)$: X → [0,1], $\eta_{A}\left(x\right)$: X → [0,1] and $\xi_{A}\left(x\right)$: X → [0,1] respectively. Fig. 5 represents the spherical fuzzy set-in three-dimensional space.

Definition 2.6 The idea of a q-SFS was introduced by Kahraman et al. [39] in the year 2020, as an extension of the existing notion of a spherical fuzzy set. Mathematically, the concept may be formally defined in the following manner.

(6) $$A=\{\left\langle x,\zeta_{A}\left(x\right)\left(x\right),\eta_{A}\left(x\right),\xi_{A}\left(x\right)\right\rangle :x∊X\}$$

**Fig. 6:**
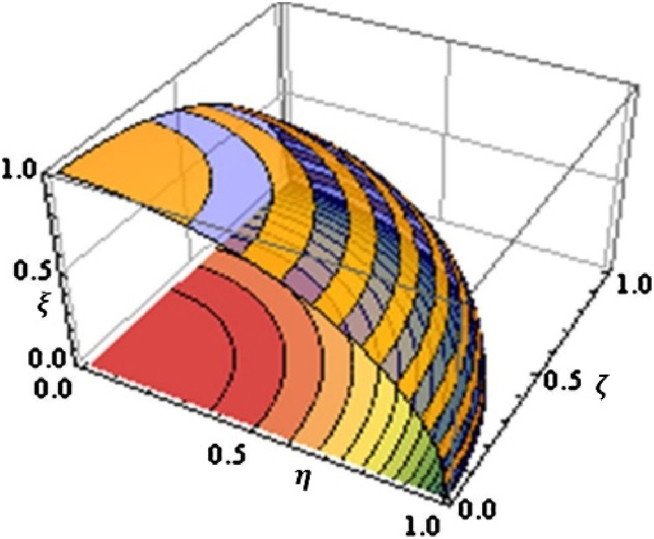
Graphical representation of q-spherical fuzzy set-in three-dimensional space.

Such that ${0\leq \left(\zeta_{A}\left(x\right)\right)}^{q}+{\left(\eta_{A}\left(x\right)\right)}^{q}+{\left(\xi_{A}\left(x\right)\right)}^{q}\leq 1$ for all q≥1. Fig. 6 represents the q-spherical fuzzy set-in three-dimensional space.

Where $\zeta_{A}$: X → [0,1], $\eta_{A}$: X → [0,1] and $\xi_{A}$: X → [0,1] correspond to the positive, neutral, and negative membership functions, respectively.

Definition 2.7 .Pawlak [40] introduced the notion of RS in back 1982. The definition of rough set is as follows: The triplet $(G_{1},G_{2},R$) is referred to as an approximation space when considering an arbitrary binary relation R on $G_{1}\times G_{2}$. The $\underline{R}\left(A\right)$ and $\bar{R}\left(A\right)$ are defined for sets X ⊆ $G_{1}$ and A⊆ $G_{2}$.

(7) $$\left(\begin{array}{c} \underline{R}\left(A\right)=\left\{x∊G_{1}:{\left[x\right]}_{A}\subseteq G\right\}\\ \bar{R}\left(A\right)=\left\{x∊G_{1}:{\left[x\right]}_{A}⋂G\neq \varphi \right\} \end{array}\right)$$

Where ${\left[x\right]}_{A}$ represents the idea of indiscernibility.

The set $\left(R\left(A\right),\bar{R}\left(A\right)\right)$ is sometimes referred to as a rough set.

Definition 2.8 [48] A q-spherical fuzzy relation R in is a q-spherical fuzzy subset of $G_{1}\times G_{2}$. and is given by

$$R=\left\{\left\langle \left(w,x\right):\zeta_{R}\left(w,x\right),{\;\eta }_{R}\left(w,x\right),{\;\xi }_{R}\left(r,s\right)\right\rangle :\left({{\left(\zeta_{R}\left(w,x\right)\right)}^{q}+\left({\;\eta }_{R}\left(w,x\right)\right)}^{q}+{\left({\;\xi }_{R}\left(w,x\right)\right)}^{q}\right)\leq 1:\forall \;w\in G_{1},x\in G_{2}\right\}\text{,}$$

where $\zeta_{R}:X\to \left[0,1\right]$, ${\;\eta }_{R}:X\to \left[0,1\right]\;\text{and}\;\xi_{R}:X\to \left[0,1\right]$.

Definition 2.9 Azim et al. [48] introduced the concept of a q-SFRS, which is defined as:

For a universal set $G_{1}\text{and}\;G_{2}\;\text{is}\;a\;\text{set}\;\text{of}\;\text{attributes}.\;\text{Let}\;R$ be a q-SF relation from $G_{1}\text{to}\;G_{2}\text{.}$ Then the triplet $(G_{1},G_{2},R$) is called q-SF approximation space. Now for any element w∈q−SFRS, the lower and upper approximation space of w w.r.t approximation space $(G_{1},G_{2},R$) are presented and given as:

(8) $$A=\left(\underline{A},\bar{A}\right)=\left\{w,\left(\begin{array}{c} {\underline{\zeta }}_{A}\left(w\right),{\underline{\zeta }}_{A}\left(w\right),{\underline{\zeta }}_{A}\left(w\right),\\ {\bar{\zeta }}_{A}\left(w\right),{\bar{\zeta }}_{A}\left(w\right),{\bar{\zeta }}_{A}\left(w\right) \end{array}\right):w\in G_{1}\right\}$$

Where,

$${\underline{\zeta }}_{A}\left(w\right)={⋀}_{x\in G_{2}}\left\{\zeta_{R}\left(w,x\right)⋀\zeta_{A}\left(x\right)\right\}\text{,}$$

$${\underline{\eta }}_{A}\left(w\right)={⋁}_{x\in G_{2}}\left\{\eta_{R}\left(w,x\right)⋁\eta_{A}\left(x\right)\right\}\text{,}$$

$${\underline{\xi }}_{A}\left(w\right)={⋁}_{x\in G_{2}}\left\{\xi_{R}\left(w,x\right)⋁\xi_{A}\left(x\right)\right\}\text{,}$$

$${\bar{\zeta }}_{A}\left(w\right)={⋁}_{x\in G_{2}}\left\{\zeta_{R}\left(w,x\right)⋁\zeta_{A}\left(x\right)\right\}\text{,}$$

$${\bar{\eta }}_{A}\left(w\right)={⋀}_{x\in U_{2}}\left\{\eta_{R}\left(w,x\right)⋀\eta_{A}\left(x\right)\right\}\text{,}$$

$${\bar{\xi }}_{A}\left(w\right)={⋀}_{x\in G_{2}}\left\{\xi_{R}\left(w,x\right)⋀\xi_{A}\left(x\right)\right\}\text{,}$$

with the condition that $\left(0\leq {{\underline{\zeta }}_{A}}^{q}\left(w\right)+{{\underline{\eta }}_{A}}^{q}\left(w\right)+{{\underline{\xi }}_{A}}^{q}\left(w\right)\leq 1\right)$ and $\left(0\leq {{\bar{\zeta }}_{A}}^{q}\left(w\right)+{{\bar{\eta }}_{A}}^{q}\left(w\right)+{{\bar{\xi }}_{A}}^{q}\left(w\right)\leq 1\right)$.

**Fig. 7:**
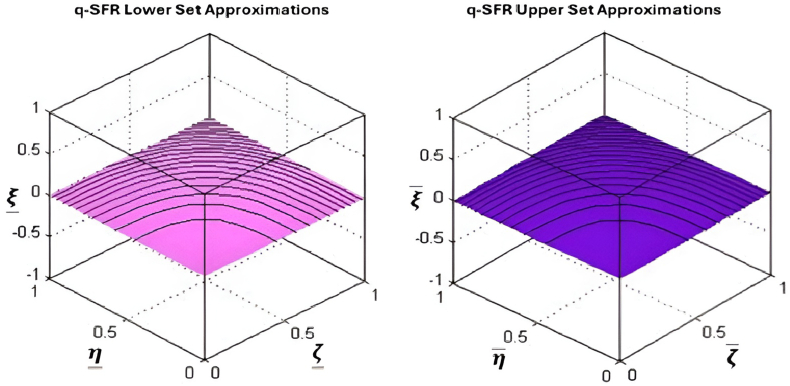
Graphical representation of q-SFRS-in three-dimensional space.

The q-SFRS is defined as a pair of q-SFSs, where $\underline{A}$ is distinct from $\bar{A}$. To facilitate comprehension, we will denote the given concept as $A=\left(\underline{A},\bar{A}\right)$, which is referred to as a q-SFRN. The notation $A_{i}$ represents the set that encompasses all q-SFR numbers. Fig. 7 represents the graphical representation of q-spherical fuzzy rough set-in three-dimensional space.

Example 2.1 Suppose there is an individual Z, who is responsible for making decisions. Currently, they are considering the purchase of a home (alternatives) from a set $G_{1}=\left\{V_{1},V_{2},V_{3},V_{4},V_{5}\right\}$. Let $G_{2}=\left\{J_{1},J_{2},J_{3},J_{4},J_{4}\right\}$ is the set of attributes. An individual seeking to make decisions Z would be interested in purchasing a home from the available alternatives in the market, prioritizing those that best meet the outlined requirements.

Consider that decision-maker Z evaluates a home's attractiveness using the q-SF relation shown in Table 1.

**Table 1:** q-Spherical fuzzy relation.

R	$J_{1}$	$J_{2}$	$J_{3}$	$J_{4}$
$V_{1}$	(0.7,0.2,0.4)	(0.5,0.2,0.7)	(0.6,0.5,0.3)	(0.7,0.2,0.5)
$V_{2}$	(0.6,0.3,0.1)	(0.3,0.2,0.5)	(0.3,0.2,0.4)	(0.5,0.2,0.5)
$V_{3}$	(0.3,0.1,0.2)	(0.7,0.4,0.3)	(0.2,0.3,0.7)	(0.3,0.4,0.1)
$V_{4}$	(0.4,0.5,0.2)	(0.8,0.4,0.5)	(0.5,0.3,0.4)	(0.4,0.3,0.1)

Imagine a decision maker, denoted as Z, who presents the best, regular decision item, denoted as A, characterized as a q-SF subset about the attribute set, to put it differently,

$$A=\{\left(J_{1}/\;\left(0.8,0.1,0.3\right)\right),\left(J_{2}/\;\left(0.8,0.4,0.3\right)\right),\left(J_{3}/\;\left(0.5,0.1,0.4\right)\right),(J_{4}/\;\left(0.5,0.4,0.6\right)\}$$

We are interested to find ${\underline{A}}_{q}$ and ${\bar{A}}_{q}$ using definition 2.6 of a set A in a relation $\left(K_{1},K_{2},R,A\right)$.

$$\underline{A}=\{\left(\frac{V_{1}}{\left(0.5,0.5,0.7\right)}\right),\left(\;\frac{V_{2}}{\left(0.3,0.4,0.6\right)}\right),\left(\frac{V_{3}}{\left(0.2,0.4,0.7\right)}\right),\left(\frac{V_{4}}{\left(0.4,0.5,0.6\right)}\right),\left(\frac{V_{5}}{\left(0.3,0.4,0.6\right)}\right)\}\text{,}$$

$$\bar{A}=\left\{\left(V_{1}/\left(0.8,0.1,0.3\right)\right),\left(\;V_{2}/\left(0.8,0.1,0.1\right)\right),\left(V_{3}\;/\left(0.8,0.1,0.1\right)\right),\left(V_{4}/\left(0.8,0.1,0.2\right)\right),\left(\;V_{5}/\left(0.9,0.1,0.1\right)\right)\right\}\text{.}$$

Therefore

$$A=\{\left(V_{1}/\left(0.5,0.5,0.7\right),\left(0.8,0.1,0.3\right)\right),\left(V_{2}/\left(0.3,0.4,0.6\right),\left(0.8,0.1,0.1\right)\right),\left(V_{3}\left(0.2,0.4,0.7\right),\left(0.8,0.1,0.1\right)\right)\text{,}$$

$$\left(V_{4}/\left(0.4,0.5,0.6\right),\left(0.8,0.1,0.2\right)\right),\left(\;V_{5}/\left(0.3,0.4,0.6\right),\left(0.9,0.1,0.1\right)\right)\}\text{.}$$

## 4 New operational laws and operators for q-SFRNs

Definition 3.1 Let $A_{1}=\left({\underline{\zeta }}_{1},{\underline{\eta }}_{1},{\underline{\xi }}_{1},{\bar{\zeta }}_{1},{\bar{\eta }}_{1},{\bar{\xi }}_{1}\right)$ and $A_{2}=\left({\underline{\zeta }}_{2},{\underline{\eta }}_{2},{\underline{\xi }}_{2},{\bar{\zeta }}_{2},{\bar{\eta }}_{2},{\bar{\xi }}_{2}\right)$ are two q-SFRNs in $(G_{1},G_{2},R$), then

$$A_{1}\cap A_{2}=\left[\left\langle \begin{array}{c} \min\left({\underline{\zeta }}_{1},{\underline{\zeta }}_{2}\right),\max\left({\underline{\eta }}_{1},{\underline{\eta }}_{2}\right),\min\left\{1-\left({\left(\min\left({\underline{\zeta }}_{1},{\underline{\zeta }}_{2}\right)\right)}^{q}+{\left(\max\left({\underline{\eta }}_{1},{\underline{\eta }}_{2}\right)\right)}^{q}\right),\min\left({\underline{\xi }}_{1},{\underline{\xi }}_{2}\right)\right\},\\ \min\left({\bar{\zeta }}_{1},{\underline{\zeta }}_{2}\right),\max\left({\bar{\eta }}_{1},{\bar{\eta }}_{2}\right),\min\left\{1-\left({\left(\min\left({\bar{\zeta }}_{1},{\underline{\zeta }}_{2}\right)\right)}^{q}+{\left(\max\left({\bar{\eta }}_{1},{\bar{\eta }}_{2}\right)\right)}^{q},\min\left({\bar{\xi }}_{1},{\bar{\xi }}_{2}\right)\right)\right\} \end{array}\right\rangle \right]$$

Definition 3.2 Let $A_{1}=\left({\underline{\zeta }}_{1},{\underline{\eta }}_{1},{\underline{\xi }}_{1},{\bar{\zeta }}_{1},{\bar{\eta }}_{1},{\bar{\xi }}_{1}\right)$ and $A_{2}=\left({\underline{\zeta }}_{2},{\underline{\eta }}_{2},{\underline{\xi }}_{2},{\bar{\zeta }}_{2},{\bar{\eta }}_{2},{\bar{\xi }}_{2}\right)$ are two q-SFRNs in $(G_{1},G_{2},R$), then

$$A_{1}\cup A_{2}=\left[\left\langle \begin{array}{c} \max\left({\underline{\zeta }}_{1},{\underline{\zeta }}_{2}\right),\min\left({\underline{\eta }}_{1},{\underline{\eta }}_{2}\right),\max\left\{1-\left({\left(\max\left({\underline{\zeta }}_{1},{\underline{\zeta }}_{2}\right)\right)}^{q}+{\left(\min\left({\underline{\eta }}_{1},{\underline{\eta }}_{2}\right)\right)}^{q}\right),\max\left({\underline{\xi }}_{1},{\underline{\xi }}_{2}\right)\right\},\\ \max\left({\bar{\zeta }}_{1},{\underline{\zeta }}_{2}\right),\min\left({\bar{\eta }}_{1},{\bar{\eta }}_{2}\right),\max\left\{1-\left({\left(\max\left({\bar{\zeta }}_{1},{\underline{\zeta }}_{2}\right)\right)}^{q}+{\left(\min\left({\bar{\eta }}_{1},{\bar{\eta }}_{2}\right)\right)}^{q}\right),\max\left({\bar{\xi }}_{1},{\bar{\xi }}_{2}\right)\right\} \end{array}\right\rangle \right]$$

Definition 3.3 Let $A_{1}=\left({\underline{\zeta }}_{1},{\underline{\eta }}_{1},{\underline{\xi }}_{1},{\bar{\zeta }}_{1},{\bar{\eta }}_{1},{\bar{\xi }}_{1}\right)$ and $A_{2}=\left({\underline{\zeta }}_{2},{\underline{\eta }}_{2},{\underline{\xi }}_{2},{\bar{\zeta }}_{2},{\bar{\eta }}_{2},{\bar{\xi }}_{2}\right)$ are two q-SFRNs in $(G_{1},G_{2},R$), then

$$A_{1}⊕A_{2}=\left[\left\langle \begin{array}{c} \sqrt[{q}]{{\underline{\zeta }}_{1}^{q}+{\underline{\zeta }}_{2}^{q}-{\underline{\zeta }}_{1}^{q}*{\underline{\zeta }}_{2}^{q}},{\;\underline{\eta }}_{1}^{q}*{\;\underline{\eta }}_{2}^{q},\sqrt[{q}]{\left(1-{\underline{\zeta }}_{2}^{q}*{\underline{\xi }}_{1}^{q}+(1-{\underline{\zeta }}_{1}^{q}*{\underline{\xi }}_{2}^{q}\right)-{\underline{\xi }}_{1}^{q}*{\underline{\xi }}_{2}^{q}}\\ ,\\ \sqrt[{q}]{{\bar{\zeta }}_{1}^{q}+{\bar{\zeta }}_{2}^{q}-{\bar{\zeta }}_{1}^{q}*{\bar{\zeta }}_{2}^{q}},{\;\bar{\eta }}_{1}^{q}*{\bar{\eta }}_{2}^{q},\sqrt[{q}]{\left(1-{\bar{\zeta }}_{2}^{q}*{\bar{\xi }}_{1}^{q}+1-{\bar{\zeta }}_{1}^{q}*{\bar{\xi }}_{2}^{q}\right)-{\bar{\xi }}_{1}^{q}*{\bar{\xi }}_{2}^{q}} \end{array}\right\rangle \right]$$

Definition 3.4 Let $A_{1}=\left({\underline{\zeta }}_{1},{\underline{\eta }}_{1},{\underline{\xi }}_{1},{\bar{\zeta }}_{1},{\bar{\eta }}_{1},{\bar{\xi }}_{1}\right)$ and $A_{2}=\left({\underline{\zeta }}_{2},{\underline{\eta }}_{2},{\underline{\xi }}_{2},{\bar{\zeta }}_{2},{\bar{\eta }}_{2},{\bar{\xi }}_{2}\right)$ are two q-SFRNs in $(G_{1},G_{2},R$), then

$$A_{1}⊗A_{2}=\left[\left\langle \begin{array}{c} {\;\underline{\zeta }}_{1}^{q}*{\underline{\zeta }}_{2}^{q},{\underline{\sqrt[{q}]{{\underline{\eta }}_{1}^{q}+{\underline{\eta }}_{2}^{q}-{\underline{\eta }}_{1}^{q}*{\underline{\eta }}_{2}^{q}}}}_{2}^{q},\sqrt[{q}]{\left(1-{\underline{\eta }}_{2}^{q}{*\underline{\xi }}_{1}^{q}+1-{\underline{\eta }}_{1}^{q}{*\underline{\xi }}_{2}^{q}\right)-{\underline{\xi }}_{1}^{q}{*\underline{\xi }}_{2}^{q}}\\ ,\\ {\;\bar{\zeta }}_{1}^{q}*{\bar{\zeta }}_{2}^{q},\sqrt[{q}]{{\bar{\eta }}_{1}^{q}+{\bar{\eta }}_{2}^{q}-{\bar{\eta }}_{1}^{q}*{\bar{\eta }}_{2}^{q}},\sqrt[{q}]{\left(1-{\bar{\eta }}_{2}^{q}{*\bar{\xi }}_{1}^{q}+1-{\bar{\eta }}_{1}^{q}{*\bar{\xi }}_{2}^{q}\right)-{\bar{\xi }}_{1}^{q}{*\bar{\xi }}_{2}^{q}} \end{array}\right\rangle \right]$$

Definition 3.5 $A=\left(\underline{\zeta },\underline{\eta },\underline{\xi },\bar{\zeta },\bar{\eta },\bar{\xi }\right)$ be any q-SFRNs in $(G_{1},G_{2},R$), and ω>0beanyscaler then,

$$\omega A=\left[\left\langle \sqrt[{q}]{{1-\left(1-{\underline{\zeta }}^{q}\right)}^{\omega }},{{\underline{\eta }}^{q}}^{\omega },\sqrt[{q}]{{\left(1-{\underline{\zeta }}^{q}\right)}^{\omega }-{\left(1-{\underline{\zeta }}^{q}-{\underline{\xi }}^{q}\right)}^{\omega }},\sqrt[{q}]{{1-\left(1-{\bar{\zeta }}^{q}\right)}^{\omega }},{{\bar{\eta }}^{q}}^{\omega },\sqrt[{q}]{{\left(1-{\bar{\zeta }}^{q}\right)}^{\omega }-{\left(1-{\bar{\zeta }}^{q}-{\bar{\xi }}^{q}\right)}^{\omega }}\right\rangle \right]$$

Definition 3.6 $A=\left(\underline{\zeta },\underline{\eta },\underline{\xi },\bar{\zeta },\bar{\eta },\bar{\xi }\right)$ be any q-SFRNs in $(G_{1},G_{2},R$), and ω>0beanyscaler then,

$$A^{\omega }=\left[\left\langle {{\underline{\zeta }}^{q}}^{\omega },\sqrt[{q}]{{1-\left(1-{\underline{\eta }}^{q}\right)}^{\omega }},\sqrt[{q}]{{\left(1-{\underline{\eta }}^{q}\right)}^{\omega }-{\left(1-{\underline{\eta }}^{q}-{\underline{\xi }}^{q}\right)}^{\omega }},{{\bar{\zeta }}^{q}}^{\omega },\sqrt[{q}]{{1-\left(1-{\bar{\eta }}^{q}\right)}^{\omega }},\sqrt[{q}]{{\left(1-{\bar{\eta }}^{q}\right)}^{\omega }-{\left(1-{\bar{\eta }}^{q}-{\bar{\xi }}^{q}\right)}^{\omega }}\right\rangle \right]$$

Definition 3.7 Let $A=\left(\underline{\zeta },\underline{\eta },\underline{\xi },\bar{\zeta },\bar{\eta },\bar{\xi }\right)$ be a q-SFRN. Then the score value which is denoted as $A_{Q\;}$ can be determined by the following function.

(9) $$Sco\left(A\right)=\frac{{2+\left(\underline{\zeta }\right)}^{q}+{\left(\bar{\zeta }\right)}^{q}-{\left(\underline{\eta }\right)}^{q}-{\left(\bar{\eta }\right)}^{q}-{\left(\underline{\xi }\right)}^{q}-{\left(\bar{\xi }\right)}^{q}}{3},q\geq 1$$

Where, 0≤Sco(A)≤1

Definition 3.8 Let $A=\left(\underline{\zeta },\underline{\eta },\underline{\xi },\bar{\zeta },\bar{\eta },\bar{\xi }\right)$ be a q-SFRN. The accuracy of A is calculated by using the formula mentioned in equation no. 10.

(10) $$Acc\left(A\right)=\frac{{\left(\underline{\zeta }\right)}^{q}+{\left(\bar{\zeta }\right)}^{q}-{\left(\underline{\xi }\right)}^{q}-{\left(\bar{\xi }\right)}^{q}}{2}$$

where −1≤Acc(A)≤1.

Definition 3.9 Let $A_{1}=\left({\underline{\zeta }}_{1},{\underline{\eta }}_{1},{\underline{\xi }}_{1},{\bar{\zeta }}_{1},{\bar{\eta }}_{1},{\bar{\xi }}_{1}\right)$ and $A_{2}=\left({\underline{\zeta }}_{2},{\underline{\eta }}_{2},{\underline{\xi }}_{2},{\bar{\zeta }}_{2},{\bar{\eta }}_{2},{\bar{\xi }}_{2}\right)$ are two q-SFRNs, then

1. If $Sco\left(A_{1}\right)<Sco\left(A_{2}\right)$ then $A_{1}<A_{2}$,
2. If $Sco\left(A_{1}\right)>Sco\left(A_{2}\right)$ then $A_{1}>A_{2}$,
3. If $Sco\left(A_{1}\right)=Sco\left(A_{2}\right)$ then
  • If $Acc\left(A_{1}\right)<Acc\left(A_{2}\right)$ then $A_{1}<A_{2}$,
  • If $Acc\left(A_{1}\right)>Acc\left(A_{2}\right)$ then $A_{1}>A_{2}$,
  • If $Acc\left(A_{1}\right)=Acc\left(A_{2}\right)$ then $A_{1}=A_{2}$.

Definition 3.10 Let $A_{1}=\left({\underline{\zeta }}_{1},{\underline{\eta }}_{1},{\underline{\xi }}_{1},{\bar{\zeta }}_{1},{\bar{\eta }}_{1},{\bar{\xi }}_{1}\right)$ and $A_{2}=\left({\underline{\zeta }}_{2},{\underline{\eta }}_{2},{\underline{\xi }}_{2},{\bar{\zeta }}_{2},{\bar{\eta }}_{2},{\bar{\xi }}_{2}\right)$ and $A=\left(\underline{\zeta },\underline{\eta },\underline{\xi },\bar{\zeta },\bar{\eta },\bar{\xi }\right)$ be any three q-SFRNs, and ω, $\omega_{1}$ and $\omega_{2}$ are any positive integers then the following properties are held.

1. $A_{1}⊕A_{2}=A_{2}⊕A_{1}$,
2. $A_{1}⊗A_{2}=A_{2}⊗A_{1}$
3. $\omega \left(A_{1}⊕A_{2}\right)=\omega A_{1}⊕\omega A_{2}$,
4. $\omega_{1}A⊕\omega_{2}A=\left(\omega_{1}+\omega_{2}\right)A$,
5. ${\left(A_{1}⊗A_{2}\right)}^{\omega }=A_{1}^{\omega }⊗A_{2}^{\omega }$,
6. $A^{\omega_{1}}⊗A^{\omega_{2}}=A^{\omega_{1}+\omega_{2}}$.

Definition 3.11 q-SFR arithmetic mean (q-SFRAM) operator concerning, $\omega =\left(\omega_{1},\omega_{2},\omega_{3},\ldots ,\omega_{n}\right)$; $\omega_{i}$ ∈ [0,1]; $\sum_{i=1}^{n}\omega_{i}=1\text{,}$ q-SFRAM operator is mathematically defined as

$${q-\text{SFRAM}}_{\omega }\left(A_{1},A_{2},A_{3},\ldots ,A_{n}\right)=\omega_{1}A_{1}⊕\omega_{2}A_{2}⊕\omega_{3}A_{3}⊕,\ldots ,{⊕\omega }_{n}A_{n}$$

$$=\left[\left\langle \begin{array}{c} \sqrt[{q}]{{\prod_{i=1}^{n}\;(1-\left(1-{\underline{\zeta }}_{i}^{q}\right)}^{\omega_{i}})},{\prod_{i=1}^{n}\;{\underline{\eta }}_{i}^{q}}^{\omega_{i}},\sqrt[{q}]{{\prod_{i=1}^{n}\;\left(1-{\underline{\zeta }}_{i}^{q}\right)}^{\omega_{i}}-{\prod_{i=1}^{n}\;\left(1-{\underline{\zeta }}_{i}^{q}-{\underline{\xi }}_{i}^{q}\right)}^{\omega_{i}}},\\ \sqrt[{q}]{{\prod_{i=1}^{n}\;(1-\left(1-{\bar{\zeta }}_{i}^{q}\right)}^{\omega_{i}})},{\prod_{i=1}^{n}\;{\bar{\eta }}_{i}^{q}}^{\omega_{i}},\sqrt[{q}]{{\prod_{i=1}^{n}\;\left(1-{\bar{\zeta }}_{i}^{q}\right)}^{\omega_{i}}-{\prod_{i=1}^{n}\;\left(1-{\bar{\zeta }}_{i}^{q}-{\bar{\xi }}_{i}^{q}\right)}^{\omega_{i}}} \end{array}\right\rangle \right]$$

Definition 3.12 q-SFR geometric mean (q-SFRGM) operator concerning, $\omega =\left(\omega_{1},\omega_{2},\omega_{3},\ldots ,\omega_{n}\right)$; $\omega_{i}$ ∈ [0,1]; $\sum_{i=1}^{n}\omega_{i}=1\text{,}$ q-SFRGM operator is mathematically defined as

$${q-\text{SFRGM}}_{\omega }\left(A_{1},A_{2},A_{3},\ldots ,A_{n}\right)=\omega_{1}A_{1}⊗\omega_{2}A_{2}⊗\omega_{3}A_{3}⊗,\ldots ,{⊗\omega }_{n}A_{n}$$

$$=\left[\left\langle \begin{array}{c} {\prod_{i=1}^{n}\;{\underline{\zeta }}_{i}^{q}}^{\omega_{i}},\sqrt[{q}]{{\prod_{i=1}^{n}\;(1-\left(1-{\underline{\eta }}_{i}^{q}\right)}^{\omega_{i}})},\sqrt[{q}]{{\prod_{i=1}^{n}\;\left(1-{\underline{\eta }}_{i}^{q}\right)}^{\omega_{i}}-{\prod_{i=1}^{n}\;\left(1-{\underline{\eta }}_{i}^{q}-{\underline{\xi }}_{i}^{q}\right)}^{\omega_{i}}},\\ {\prod_{i=1}^{n}\;{\bar{\zeta }}_{i}^{q}}^{\omega_{i}},\sqrt[{q}]{{\prod_{i=1}^{n}\;(1-\left(1-{\bar{\eta }}_{i}^{q}\right)}^{\omega_{i}})},\sqrt[{q}]{{\prod_{i=1}^{n}\;\left(1-{\bar{\eta }}_{i}^{q}\right)}^{\omega_{i}}-{\prod_{i=1}^{n}\;\left(1-{\bar{\eta }}_{i}^{q}-{\bar{\xi }}_{i}^{q}\right)}^{\omega_{i}}} \end{array}\right\rangle \right]$$

## 5 q-spherical fuzzy rough TOPSIS

TOPSIS, an acronym for "Technique for Order Preference by Similarity to Ideal Solution," is a decision-making methodology commonly employed in multicriteria decision analysis. It serves as a valuable tool for selecting the optimal choice from a pool of alternatives when multiple criteria are taken into consideration during the decision-making process. Because it requires some degree of resemblance to the ideal answer to be effective, this makes it a very potent strategy in certain situations. Yoon and Ching-Lai Hwang originally presented the TOPSIS approach in 1981, and Yoon improved it again in 1987. This technique ranks several alternatives based on how near they are to the ideal solution to determine which compromise solution is the most desirable. Distance measures are used in this selection procedure to weigh and choose possibilities. The core notion of traditional TOPSIS approaches is to find the optimal solution that strikes a compromise between being close to the positive ideal solution and being distant from the negative ideal solution. Experts in decision-making have devoted a great deal of work to studying the TOPSIS technique throughout the years. These studies have demonstrated the effectiveness with which TOPSIS may be utilized in a range of specialized domains. Decision-makers can assess and rank different options using the TOPSIS approach, which stands for the Order of Preference by Similarity to Ideal Solution. They can then use data to support their well-informed judgments. When weighing alternatives, decision-makers may consider a variety of criteria and their relative importance thanks to TOPSIS's methodical framework. It provides a methodical approach to handling intricate circumstances with several elements that must be considered at the same time. By calculating the distance between the options and the optimal answer, a decision-making technique known as TOPSIS helps determine which option is the most favorable. It allows decision-makers to find a balance between desired attributes and objectives. In summary, TOPSIS is a well-regarded and effective technique for Multi-Criteria Decision Making. The practicality, usefulness, and capability to handle intricate decision problems have resulted in its widespread adoption by both researchers and practitioners.

A decision matrix is a method used to represent a multi-criteria decision-making (MCDM) problem. The elements of the decision matrix represent the evaluation scores assigned to various possibilities for each criterion inside a system functioning within a q-spherical fuzzy rough framework. Let X be a discrete collection of m possible alternatives, denoted as $X=\left\{x_{1},x_{2},x_{3},\ldots ,x_{m}\right\}$, where m is greater than or equal to 2. The present collection contains the many values associated with each criterion, considering all possible alternatives, within the context of a q-SFR model. In the context of this q-SFR environment, we examine a collection of criteria represented by the set $J=\left\{J_{1},J_{2},J_{3},\ldots ,J_{n}\right\}$. The weight vector for all criteria is denoted as W, subject to the restrictions $0\leq w_{i}\leq 1$ and $\sum_{i=1}^{n}w_{i}=1$. It is represented as $W=\left\{w_{1},w_{2},w_{3},\ldots ,w_{n}\right\}$.

Step 1 The individual responsible for decision-making uses the linguistic terms provided in Table 1 to complete the assessment matrix.

Step 2 The decision matrix is transformed into a weighted q-spherical fuzzy rough decision matrix by using the q-spherical fuzzy rough values presented in Table 1. The derivation of the weighted q-spherical fuzzy rough decision matrix involves the multiplication of criteria weights with the evaluations. The matrix computation is derived using the specified formula in Definition 3.4.

Step 3 The process of defuzzifying the weighted decision matrix entails the application of the specified score function, which may be implemented using the formula shown below.

$$Sco\left(A\right)=\frac{{2+\left(\underline{\zeta }\right)}^{q}+{\left(\bar{\zeta }\right)}^{q}-{\left(\underline{\eta }\right)}^{q}-{\left(\bar{\eta }\right)}^{q}-{\left(\underline{\xi }\right)}^{q}-{\left(\bar{\xi }\right)}^{q}}{3},q\geq 1$$

Step 4 The q-SFR Positive Ideal Solution (q-SFRPIS) and q-SFR Negative Ideal Solution (q-SFRNIS) are constructed using the step 3 score values. Regarding the q-SFRPIS:

$$X^{*}=\left\{C_{j},\max_{i}<Score\left(C_{j}\left(X_{iw}\right)\right)>j=1,2,\ldots ,n\right\}$$

For the q-SFRNIS:

$$X^{-}=\left\{C_{j},\min_{i}<Score\left(C_{j}\left(X_{iw}\right)\right)>j=1,2,\ldots ,n\right\}$$

Step 5 q-SFRPIS and q-SFRNIS are derived from the scores acquired in step 3. With regards to q-SFRPIS:

$$D\left(X_{i},X^{-}\right)=\sqrt[{q}]{\frac{1}{4n}\sum_{i=1}^{n}\left({\left({{\underline{\zeta }}_{x_{i}}}^{q}-{{\underline{\zeta }}_{x^{-}}}^{q}\right)}^{2}+{\left({{\bar{\zeta }}_{x_{i}}}^{q}-{{\bar{\zeta }}_{x^{-}}}^{q}\right)}^{2}+{\left({{\underline{\eta }}_{x_{i}}}^{q}-{{\underline{\eta }}_{x^{-}}}^{q}\right)}^{2}+{\left({{\bar{\eta }}_{x_{i}}}^{q}-{{\bar{\eta }}_{x^{-}}}^{q}\right)}^{2}+{\left({{\underline{\xi }}_{x_{i}}}^{q}-{{\underline{\xi }}_{x^{-}}}^{q}\right)}^{2}{+\left({{\bar{\xi }}_{x_{i}}}^{q}-{{\bar{\xi }}_{x^{-}}}^{q}\right)}^{2}\right)}$$

For the q-SFRPIS:

$$D\left(X_{i},X^{*}\right)=\sqrt[{q}]{\frac{1}{4n}\sum_{i=1}^{n}\left({\left({{\underline{\zeta }}_{x_{i}}}^{q}-{{\underline{\zeta }}_{x^{*}}}^{q}\right)}^{2}+{\left({{\bar{\zeta }}_{x_{i}}}^{q}-{{\bar{\zeta }}_{x^{*}}}^{q}\right)}^{2}+{\left({{\underline{\eta }}_{x_{i}}}^{q}-{{\underline{\eta }}_{x^{*}}}^{q}\right)}^{2}+{\left({{\bar{\eta }}_{x_{i}}}^{q}-{{\bar{\eta }}_{x^{*}}}^{q}\right)}^{2}+{\left({{\underline{\xi }}_{x_{i}}}^{q}-{{\underline{\xi }}_{x^{*}}}^{q}\right)}^{2}{+\left({{\bar{\xi }}_{x_{i}}}^{q}-{{\bar{\xi }}_{x^{*}}}^{q}\right)}^{2}\right)}$$

Step 6 Several computations are carried out to establish the minimum and maximum distances to both the q-SFRPIS and the q-SFRNIS. These distances are computed as follows:

Dmax(Xi,X−)=D(Xi,X−)1≤i≤mmax

Dmin(Xi,X*)=D(Xi,X*)1≤i≤mmin

Step 7 When calculating the closeness ratio, the revised closeness ratio that was recommended by (KutluGündoğdu and Kahraman, 2019) is utilized. This ratio is used in the calculation.

$$₯\left(X_{i}\right)=\frac{D\left(X_{i},X^{*}\right)}{D_{\min}\left(X_{i},X^{*}\right)}-\frac{D\left(X_{i},X^{-}\right)}{D_{\max}\left(X_{i},X^{-}\right)}$$

Step 8 The closeness ratio is used to rank the possible alternatives in descending order.

**Fig. 8:**
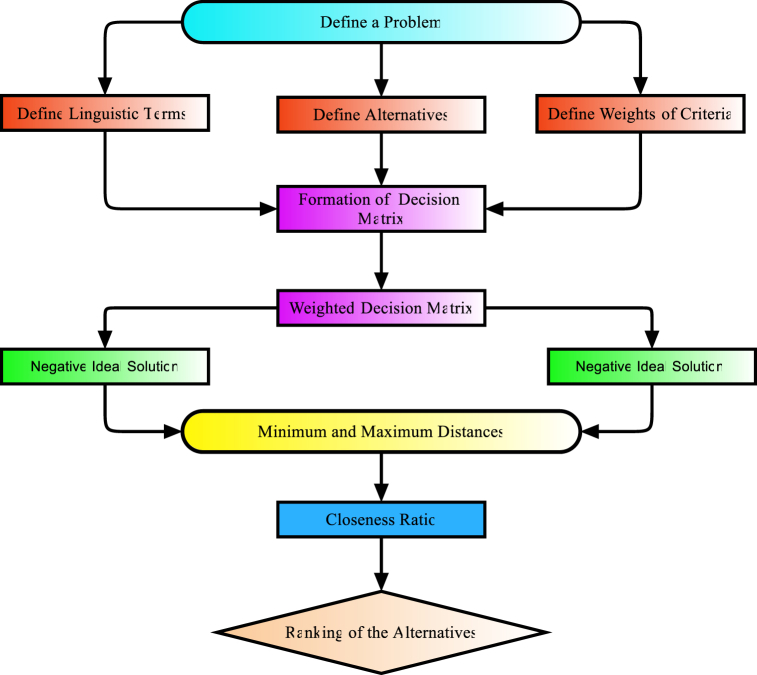
Typical framework of the multi-criteria decision-making approach.

Fig. 8 shows the multi-criteria decision-making (MCDM) process in a format that is commonly used. This structure, as shown in Fig. 8, typically consists of the following steps: defining criteria, giving these criterion weights, and adding up the scores to determine which selection is best.

## 6 .Decision model and applications

**Fig. 9:**
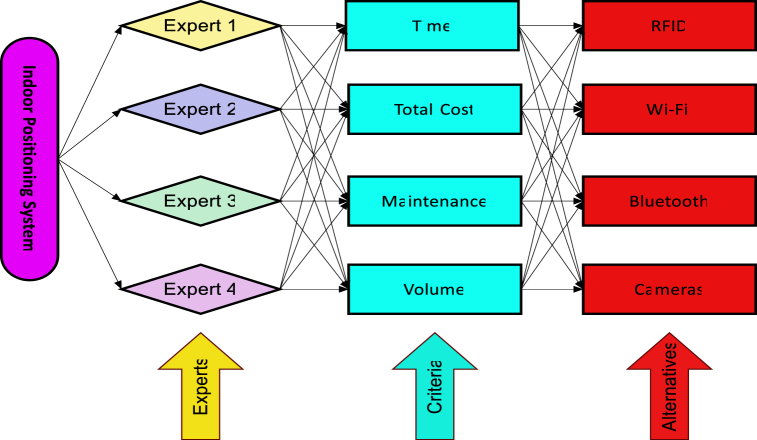
Decision tree for the assessing indoor positioning system.

When it comes to analyzing the behavior of customers in indoor locations, many different approaches may be used to gather data. The following are some examples of different types of alternative technologies: RFID $V_{1}$, or radio frequency identification, is a technology that, when applied to things that are being tracked, enables those objects to link with radio frequency readers using tags that are affixed to the objects. Wi-Fi $V_{2}$, is another kind of technology that has applications inside of buildings Messages with a singular identifier are regularly sent by electronic gadgets that are equipped with Wi-Fi capabilities. Once these communications have been retrieved by Wi-Fi scanners, the subsequent stage in the procedure may then be carried out. If each device has its own distinct identity, it will be much simpler to locate and keep track of all the devices. Bluetooth $V_{3}$, is another kind of technology that may be used to keep track of a person's location inside. Signals that are delivered by Bluetooth contain the Bluetooth media access control address, which may be used again to identify the device. This address can be found in the Bluetooth header. As a result of this, when enough scanners are deployed, it will be possible to identify the position of the object with a high degree of accuracy. The position tracking system is completed with the addition of the video cameras $V_{4}$, as the last component. To produce a description of the object that is to be observed, this approach makes use of sophisticated image processing algorithms that have elaborate architectures. We produced a range of alternative evaluation criteria because of conducting interviews with subject matter experts and conducting a throughout literature review; however, for this research, we concentrated on choosing the four criteria that were the most important. The quantity of "Time" needed to gather and analyze data is reflected in the value of the variable $J_{1}$, which is also known as time. The criterion that is denoted by the letter $J_{2}$ and is referred to as "Total Cost" which reflects the total cost that must be expended to collect the data. The processes that are carried out to ensure that the system continues to function appropriately are referred to as “Maintenance” and are represented by the letter $J_{3}\text{.}$ In addition to this, other requirements pertain to the data itself, including its actual content. For instance, the amount of data that was obtained by using the technology is shown by the criteria known as "Volume," which is represented by the symbol $J_{4}$. This information was obtained. Fig. 9 illustrates a decision tree used for assessing indoor positioning systems Table 2.

The experts who oversaw the decision consider the evaluation, which is shown in Table 3.

**Table 2:** Linguistic terms in conjunction with their associated q-SFR numbers.

Linguistic Terms	$\left(\underline{\mu },\underline{\nu },\underline{\pi }\right)$	$\left(\bar{\mu },\bar{\nu },\bar{\pi }\right)$
Absolutely More Importance (AMI)	(0.95.0.55,0.55)	(0.91,0.51,0.51)
Very High Importance (VHI)	(0.90,0.60,0.60)	(0.86,0.56,0.56)
High Importance (HI)	(0.85,0.65,0.65)	(0.81,0.61,0.61)
Slightly More Importance (SMI)	(0.80,0.70,0.70)	(0.76,0.66,0.66)
Importance (EI)	(0.75,0.75,0.75)	(0.71,0.71,0.71)
Slightly Low Importance (SLI)	(0.70,0.80,0.70)	(0.66,0.76,0.66)
Low Importance (LI)	(0.65,0.85,0.65)	(0.61,0.81,0.61)
Very Low Importance (VLI)	(0.60,0.90,0.60)	(0.56,0.86,0.56)
Absolutely Low Importance (ALI)	(0.55,0.95,0.55)	(0.51,0.91,0.51)

**Table 3:** Evaluation of the alternatives for indoor location tracking.

Alternatives	$J_{1}$	$J_{2}$	$J_{3}$	$J_{4}$
$V_{1}$	SMI	HI	LI	SMI
$V_{2}$	EI	HI	HI	HI
$V_{3}$	HI	SMI	SMI	SMI
$V_{4}$	SMI	VHI	LI	VHI

The assessment undergoes a conversion process into q-SFRSs, following the method outlined in the methodology section, as illustrated in Table 4.

**Table 4:** q-SFR information.

**Alternatives**	$J_{1}$	$J_{2}$	$J_{3}$	$J_{4}$
$V_{1}$	$\left(\begin{array}{c} \left(0.80,0.70,0.70\right)\text{,}\\ \left(0.76,0.66,0.66\right) \end{array}\right)$	$\left(\begin{array}{c} \left(0.85,0.65,0.65\right)\text{,}\\ \left(0.81,0.61,0.61\right) \end{array}\right)$	$\left(\begin{array}{c} \left(0.65,0.85,0.65\right)\text{,}\\ \left(0.61,0.81,0.61\right) \end{array}\right)$	$\left(\begin{array}{c} \left(0.80,0.70,0.80\right)\text{,}\\ \left(0.76,0.66,0.66\right) \end{array}\right)$
$V_{2}$	$\left(\begin{array}{c} \left(0.75,0.75,0.75\right)\text{,}\\ \left(0.71,0.71,0.71\right) \end{array}\right)$	$\left(\begin{array}{c} \left(0.85,0.65,0.65\right)\text{,}\\ \left(0.81,0.61,0.61\right) \end{array}\right)$	$\left(\begin{array}{c} \left(0.85,0.65,0.65\right)\text{,}\\ \left(0.81,0.61,0.61\right) \end{array}\right)$	$\left(\begin{array}{c} \left(0.85,0.65,0.65\right)\text{,}\\ \left(0.81,0.61,0.61\right) \end{array}\right)$
$V_{3}$	$\left(\begin{array}{c} \left(0.85,0.65,0.65\right)\text{,}\\ \left(0.81,0.61,0.61\right) \end{array}\right)$	$\left(\begin{array}{c} \left(0.80,0.70,0.70\right)\text{,}\\ \left(0.76,0.66,0.66\right) \end{array}\right)$	$\left(\begin{array}{c} \left(0.80,0.70,0.70\right)\text{,}\\ \left(0.76,0.66,0.66\right) \end{array}\right)$	$\left(\begin{array}{c} \left(0.80,0.70,0.70\right)\text{,}\\ \left(0.76,0.66,0.66\right) \end{array}\right)$
$V_{4}$	$\left(\begin{array}{c} \left(0.80,0.70,0.70\right)\text{,}\\ \left(0.76,0.66,0.66\right) \end{array}\right)$	$\left(\begin{array}{c} \left(0.90,0.60,0.60\right)\text{,}\\ \left(0.86,0.56,0.56\right) \end{array}\right)$	$\left(\begin{array}{c} \left(0.65,0.85,0.65\right)\text{,}\\ \left(0.61,0.81,0.61\right) \end{array}\right)$	$\left(\begin{array}{c} \left(0.90,0.60,0.60\right)\text{,}\\ \left(0.86,0.56,0.56\right) \end{array}\right)$

The next step is to figure out how to make a weighted decision matrix. The person making the decision picks the weights of the criteria, which are shown in Table 5.

**Table 5:** Weights of the criteria.

Lower and upper set approximation	$w_{1}$	$w_{2}$	$w_{3}$	$w_{4}$
$\left(\underline{\mu },\underline{\nu },\underline{\pi }\right)$	(0.30,0.10,0.10)	(0.30,0.20,0.20)	(0.20,0.30,0.30)	(0.20,0.40,0.40)
$\left(\bar{\mu },\bar{\nu },\bar{\pi }\right)$	(0.30,0.40,0.10)	(0.30,0.20,0.20)	(0.20,0.10,0.30)	(0.20,0.30,0.40)

The weights are multiplied by the decision matrix to make the weighted q-SFR decision matrix. Table 6 shows this.

**Table 6:** Weighted q-SFR decision matrix.

**Alternatives**	$J_{1}$	$J_{2}$	$J_{3}$	$J_{4}$
$V_{1}$	$\left(\begin{array}{c} \left(0.013,0.700,0.700\right)\text{,}\\ \left(0.011,0.693,0.645\right) \end{array}\right)$	$\left(\begin{array}{c} \left(0.016,0.654,0.651\right)\text{,}\\ \left(0.014,0.628,0.608\right) \end{array}\right)$	$\left(\begin{array}{c} \left(0.002,0.854,0.646\right)\text{,}\\ \left(0.001,0.810,0.615\right) \end{array}\right)$	$\left(\begin{array}{c} \left(0.004,0.727,0.698\right)\text{,}\\ \left(0.003,0.664,0.678\right) \end{array}\right)$
$V_{2}$	$\left(\begin{array}{c} \left(0.014,0.750,0.749\right)\text{,}\\ \left(0.009,0.736,0.694\right) \end{array}\right)$	$\left(\begin{array}{c} \left(0.016,0.654,0.651\right)\text{,}\\ \left(0.014,0.628,0.608\right) \end{array}\right)$	$\left(\begin{array}{c} \left(0.004,0.665,0.653\right)\text{,}\\ \left(0.004,0.610,0.622\right) \end{array}\right)$	$\left(\begin{array}{c} \left(0.004,0.684,0.658\right)\text{,}\\ \left(0.004,0.615,0.638\right) \end{array}\right)$
$V_{3}$	$\left(\begin{array}{c} \left(0.016,0.650,0.650\right)\text{,}\\ \left(0.014,0.651,0.597\right) \end{array}\right)$	$\left(\begin{array}{c} \left(0.013,0.703,0.699\right)\text{,}\\ \left(0.011,0.674,0.656\right) \end{array}\right)$	$\left(\begin{array}{c} \left(0.80,0.70,0.70\right)\text{,}\\ \left(0.76,0.66,0.66\right) \end{array}\right)$	$\left(\begin{array}{c} \left(0.004,0.727,0.698\right)\text{,}\\ \left(0.003,0.664,0.678\right) \end{array}\right)$
$V_{4}$	$\left(\begin{array}{c} \left(0.013,0.700,0.700\right)\text{,}\\ \left(0.011,0.693,0.645\right) \end{array}\right)$	$\left(\begin{array}{c} \left(0.019,0.605,0.602\right)\text{,}\\ \left(0.017,0.582,0.560\right) \end{array}\right)$	$\left(\begin{array}{c} \left(0.65,0.85,0.65\right)\text{,}\\ \left(0.61,0.81,0.61\right) \end{array}\right)$	$\left(\begin{array}{c} \left(0.005,0.643,0.620\right)\text{,}\\ \left(0.005,0.566,0.599\right) \end{array}\right)$

The subsequent phase entails the establishment of both the negative and positive ideal q-SFR values. The process of achieving this involves the application of the score function to defuzzify the q-SFR values. The outcomes are showcased in Table 7, as illustrated hereunder.

**Table 7:** The defuzzified values of the q-spherical fuzzy rough decision matrix.

**Alternatives**	$J_{1}$	$J_{2}$	$J_{3}$	$J_{4}$
$V_{1}$	0.2370	0.3235	0.1134	0.2228
$V_{2}$	0.1406	0.3235	0.3191	0.2999
$V_{3}$	0.2785	0.3180	0.0675	0.2994
$V_{4}$	0.2786	0.3167	0.2825	0.2892

The PIS and NIS values are calculated with the help of the information supplied in Table 7, which is followed by the information displayed in Table 8, which concludes the process.

**Table 8:** PIS and NIS of alternatives.

	$J_{1}$	$J_{2}$	$J_{3}$	$J_{4}$
PIS	$\left(\begin{array}{c} \left(0.013,0.650,0.700\right)\text{,}\\ \left(0.011,0.651,0.645\right) \end{array}\right)$	$\left(\begin{array}{c} \left(0.016,0.654,0.651\right)\text{,}\\ \left(0.014,0.628,0.608\right) \end{array}\right)$	$\left(\begin{array}{c} \left(0.004,0.665,0.653\right)\text{,}\\ \left(0.004,0.610,0.622\right) \end{array}\right)$	$\left(\begin{array}{c} \left(0.004,0.684,0.658\right)\text{,}\\ \left(0.004,0.615,0.638\right) \end{array}\right)$
NIS	$\left(\begin{array}{c} \left(0.011,0.750,0.749\right),\\ \left(0.011,0.750,0.749\right) \end{array}\right)$	$\left(\begin{array}{c} \left(0.019,0.703,0.602\right)\text{,}\\ \left(0.017,0.674,0.560\right) \end{array}\right)$	$\left(\begin{array}{c} \left(0.004,0.854,0.699\right)\text{,}\\ \left(0.003,0.810,0.668\right) \end{array}\right)$	$\left(\begin{array}{c} \left(0.004,0.727,0.698\right)\text{,}\\ \left(0.003,0.664,0.678\right) \end{array}\right)$

**Table 9:** The distance of each alternative from the PIS and NIS values.

Alternatives	Positive ideal solution	Negative ideal solution
$V_{1}$	0.2407	0.1404
$V_{2}$	0.1434	0.2453
$V_{3}$	0.1473	0.2450
$V_{4}$	0.2435	0.1926

According to the data shown in Table 8, the maximum distance to the q-SFRNIS is 0.2453, Table 9 and the minimum distance to the q-SFRPIS is 0.1284. In the last step of the procedure, the closeness ratio is computed making use of Step 7, which can be found in Table 10.

**Table 10:** The closeness ratio and the rank of the alternatives.

Alternatives	Closeness ratio	Ranking order
$V_{1}$	1.1062	4
$V_{2}$	0.0000	1
$V_{3}$	0.0284	2
$V_{4}$	0.9129	3

**Fig. 10:**
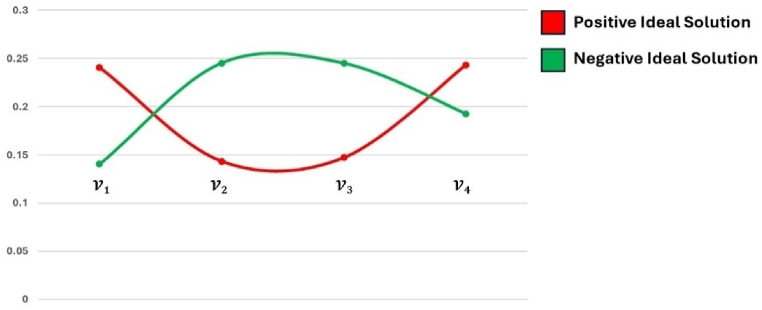
Graphical Representation of positive ideal solution and negative ideal solution.

The graphical representation of positive ideal solution and negative ideal solution is represented in Fig. 10.

**Fig. 11:**
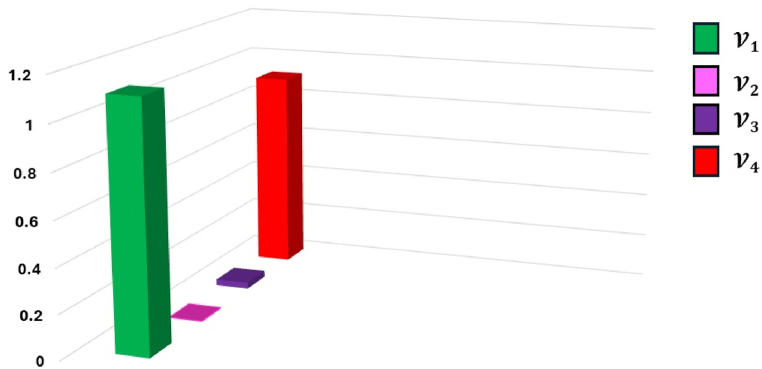
Graphical representation of closeness ratio.

Fig. 11 displays a graphical representation of the closeness ratio ranking.

We have concluded that alternative $V_{2}$ (Wi-Fi), which has the lowest closeness ratio, is the most convenient alternative because of the results of the study, and we have come to this conclusion based on the data. This alternative is followed by $V_{3}$ (Bluetooth), ${\;V}_{4}$ (Video Camera) and $V_{1}$ (RFID). From the previous discussion, it is evident that the existing aggregation operators might be viewed as special instances within the suggested framework. This conclusion suggests that the suggested method offers a more comprehensive and wide-ranging approach compared to the current aggregation operators. From the previous discussion, it is evident that the existing aggregation operators might be viewed as special instances within the suggested framework. This conclusion suggests that the suggested method offers a more comprehensive and wide-ranging approach compared to the current aggregation operators. Furthermore, the information demonstrated that, even with the recommended operators, the alternatives' ranking order does not shift. This suggests that any of these operators can be applied at any point throughout the aggregate process without having a substantial impact on the final ranking of the alternatives. Therefore, additional considerations like processing speed or requirements in the decision-making setting could have an impact on the operator's choice.

### 6.1 Effect of q ranking order and score values

To fulfill the constraint requirement $\left(0\leq {{\underline{\zeta }}_{A}}^{q}\left(w\right)+{{\underline{\eta }}_{A}}^{q}\left(w\right)+{{\underline{\xi }}_{A}}^{q}\left(w\right)\leq 1\right)$ and $\left(0\leq {{\bar{\zeta }}_{A}}^{q}\left(w\right)+{{\bar{\eta }}_{A}}^{q}\left(w\right)+{{\bar{\xi }}_{A}}^{q}\left(w\right)\leq 1\right)$, and then by examining the attribute values, a decision-maker can determine which integer parameter, q, is the smallest. For example, while evaluating an alternative, if the attribute values are (0.8,0.7,0.9,0.9,0.8,0.7), one should choose q as 3 or q as 4, as both configurations meet the criterion. However, we employed several values of q in Step 4 of the novel approach to solve the case to fully evaluate the effect of parameter q on the experimental results. The results indicate that $V_{2}$ is at the top, followed by $V_{3}$, $V_{4}$, and finally, $V_{1}$. The ranking is the same for all q values i.e. $V_{2}>V_{3}>V_{4}>V_{1}$. This consistent ranking provides decision-makers with a robust framework to evaluate test alternatives within a given collection of finite alternatives. This gives decision-makers a secure and adaptable environment, facilitating careful examination and well-informed choices based on the specified parameters.

#### 6.1.1 Test of validity

To illustrate the adaptability of the proposed technique in various settings, we utilize the evaluation protocols developed by Wang and Trianaphyllou [59] in the following ways.

Step 1 Replacing the rating values of less-than-ideal alternatives with those of inferior quality shouldn't affect the identification of the best alternative, preserving the selection that is rated highest, and assuming stable relative weights for the criterion.

Step 2 Transitivity should be followed in the procedure.

Step 3 When using the same decision-making process for a given problem that has been broken into smaller ones, the initial ranking of the alternatives should be preserved.

### 6.2 Test of validity utilizing criteria 1

The alternatives ranked by using our suggested method are $V_{2}>V_{3}>V_{4}>V_{1}$. Based on test criteria 1, we replaced the non-optimal alternative $V_{1}$ with the lowest alternative $V_{1}^{*}$ to evaluate the stability of the suggested method. ((0.90,0.60,0.60),(0.86,0.56,0.56)), ((0.80,0.70,0.70),(0.76,0.66,0.66)) and ((0.60,0.90,0.60),(0.56,0.86,0.56)) were used as the rating values of $V_{1}^{*}\text{.}$ The aggregated score values for the alternatives were as follows after we used our suggested methodology: $Sco\left(V_{1}^{*}\right)=0.6524$, $Sco\left(V_{2}\right)=0.8390$, $Sco\left(V_{3}\right)=0.7245$, and $Sco\left(V_{4}\right)=0.7165$. As a result, $V_{2}>V_{3}>V_{4}>V_{1}^{*}$ is the new ranking order, and the best alternative still adheres to the first suggested strategy. Consequently, our method meets test requirement 1 by producing a consistent result.

### 6.3 Test of validity employing criteria 1 and 2

The fragmented decision-making subcases are regarded as $\left\{V_{1},V_{2},V_{3}\right\}$, $\left\{V_{2},V_{3},V_{4}\right\}$ and $\left\{V_{1},V_{3},V_{4}\right\}$ to assess the validity based on criteria 1 and 2. They rank in the following sequence via the procedures mentioned: $V_{2}>V_{3}>V_{1}$, $V_{2}>V_{3}>V_{4}$ and $V_{3}>V_{4}>V_{1}$. After combining all the findings, the overall ranking appears as $V_{2}>V_{3}>V_{4}>V_{1}$, which is exactly in line with the outcomes of the initial decision-making process. As a result, our suggested strategy meets requirements 1 and 2.

## 7 .Managerial implications

The advent of the innovative q-SFR TOPSIS model bears significant managerial implications. It plays a crucial role in aiding managers and decision-makers in shaping strategic choices and attaining robust, reliable results. This framework demonstrates impressive versatility across a range of industries and proves to be effective in diverse decision-making situations. Managers from various sectors can efficiently harness the potential of the q-SFR TOPSIS model for a multitude of purposes. I offer this paraphrasing to ensure originality and authenticity in the content. For example, it demonstrates its worth in the process of indoor location tracking selection by assisting in the evaluation of various factors to identify the most advantageous indoor location tracking technology. Moreover, it can aid in the selection of maintenance strategies, allowing managers to choose the most suitable maintenance approach for their equipment or systems. The assessment of robots in industrial settings is another field where the model can be utilized, aiding managers in evaluating the effectiveness and appropriateness of various robotic solutions. Additionally, it can be employed in the process of selecting material handling equipment, assisting managers in making informed decisions about the most optimal and productive equipment that suits their unique needs. Nevertheless, it is crucial to acknowledge that the decision-making process within this framework is dependent on the preferences of experts and individuals who are engaged in it. The model offers a methodical and organized method for decision-making, but the ultimate decisions and rankings will ultimately be based on the judgments and preferences of the decision-makers. Therefore, it becomes imperative to involve experts and stakeholders to ensure the precision and significance of the findings. To ensure the credibility and resilience of the obtained results, two pivotal analyses are conducted.

## 8 Comparative analysis

The analysis serves as a valuable tool for decision-makers in assessing and comparing rankings and outcomes among different alternatives, each evaluated based on distinct criteria, IT enables a deeper understanding of trad0offs and facilitates well-informed decision-making by highlighting the strengths and weaknesses of each alternative.

## 9 Sensitivity analysis

By doing so, it offers crucial insights into the stability and sensitivity of the outcomes. Decision-makers can thus evaluate how various factors influence their choices, enhancing their ability to make adaptive decisions in dynamic environments. By incorporating this analysis into the decision-making process, managers can enhance the reliability and confidence in their strategic decisions. The q-SFR TOPSIS model, in conjunction with comparative and sensitivity analysis, provides a comprehensive framework that equips managers across diverse industries and applications with the tools needed to make informed and resilient decisions.

### 9.1 Comparative analysis

When examining methodologies for selecting flexible manufacturing systems, various approaches have been proposed, each offering distinct advantages. Rao and Parnichkun [60] advocate for a combinatorial mathematics-based decision-making method, while Maniya and Bhatt [61] introduce the preference selection index method, catering to different decision-making preferences and requirements. Karande and Chakraborty [62] utilize the MACBETH method, emphasizing a systematic evaluation and selection process. Moreover, Mathew and Thomas [63] explore interval-valued multi-criteria decision-making methods, which could potentially accommodate uncertainties in decision criteria. In a broader scope, the research by Hayat et al. [64] on new aggregation operators for group-based generalized intuitionistic fuzzy soft sets, and Yang et al.'s [65] work on aggregation operators for interval-valued q-Rung Orthopair fuzzy soft environment with applications in automation company evaluation, underscore the importance of innovative aggregation techniques in handling complex and diverse data structures. Additionally, Mehmood et al. [66] propose a multi-criteria decision-making method for cubic hesitant fuzzy sets based on Einstein's operational laws. Drawing parallels, the proposed q-spherical fuzzy rough TOPSIS method could provide a comprehensive framework for decision-making in scenarios involving flexible manufacturing system selection. By integrating fuzzy rough set theory with TOPSIS, it offers a robust approach to dealing with imprecise, uncertain, and incomplete information, mirroring the challenges often encountered in real-world decision-making processes. Thus, it stands as a promising avenue for enhancing the effectiveness and reliability of decision-making in this domain, bridging the gap between theoretical advancements and practical applications in indoor locating system selection. Table 11 represents the comparative analysis of different methodologies with the proposed operator.

**Table 11:** Comparative analysis of rankings across differing methodologies.

Alternatives	**Rao et. al** [60]	**Maniya et. al** [61]	**Karande et. al [69]**	**Mathew et. al** [63]	**Hayat et. al** [64]	**Yang et. al** [65]	**Mehmood et. al** [66]	**Azim et. al [Proposed]**
$V_{1}$	2	2	2	2	3	3	1	4
$V_{2}$	1	1	1	1	2	4	3	1
$V_{3}$	3	3	4	4	4	2	2	2
$V_{4}$	4	4	3	3	1	1	×	3

**Fig. 12:**
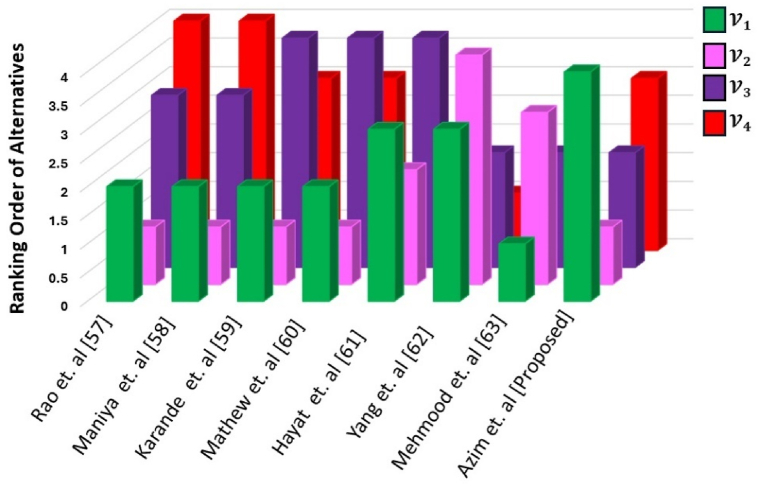
Visual representation of the rankings acquired using different methodologies.

Fig. 12 presents a visual representation of the rankings acquired using different methodologies. The alternatives that look highest in the bar graph mean that it is the lowest in ranking.

### 9.2 Sensitivity analysis

**Fig. 13:**
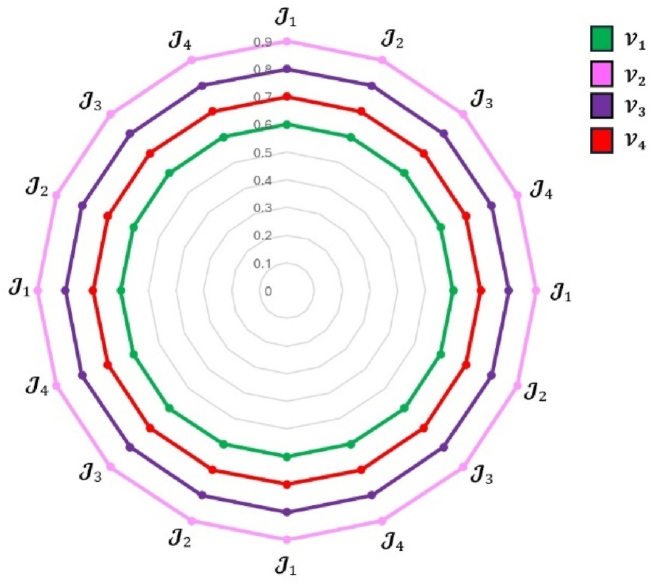
Alternative classification considering variations in criteria weights.

In this work, the developed model is verified using two separate sensitivity analyses that focus on changes in criteria and decision-making weights and examine their impact on final rankings. In the first research, a temporal sensitivity analysis is undertaken to explore the effect of varying the weights of reference criteria with high, equal, and low priority on the overall ranking. The model is then run separately for each criterion, assigning reference weights one at a time. Fig. 13 displays the results from twenty distinct settings. In all circumstances, alternative $V_{2}$ consistently ranks first, while alternative $V_{1}$ regularly ranks last. Notably, despite considerable variations in the criterion weights, the model output is quite insensitive.

**Fig. 14:**
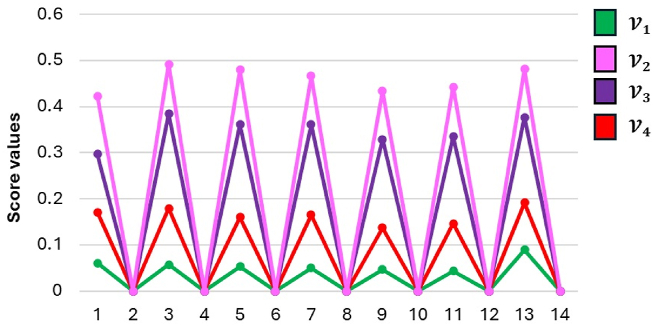
Alternative rankings in response to adjustments in decision-maker's weights.

The second research focuses on adjusting the weights assigned to decision-makers, resulting in four possible situations with different weight distributions. Fig. 14 displays the final rankings for these scenarios. In all scenarios, alternative $V_{2}$ is consistently the greatest alternative, whereas alternative $V_{1}$ is continuously the least loved. Although the relative rank of the alternatives differs according to the decision-maker weights utilized, the proposed approach is robust and consistent over a wide range of decision-weighting scenarios.

### 9.3 Advantages

The proposed technique has various benefits.

1. The addition of parameter q to the aggregation operators gives decision-makers a great deal of freedom. This versatility allows them to tailor the settings to the individual needs and preferences of the decision-making scenarios. The decision process's versatility allows for varying degrees of membership, neutral membership, and non-membership with lower and upper set approximations, making it appropriate for a broad range of real-world scenarios.
2. The parametric character of the suggested operators enables decision-makers to fine-tune the impact of membership, neutral membership, and non-membership degrees with lower and upper set approximations. This degree of control enables decision-makers to accurately tailor the aggregation process to their preferences and the unique aspects of the situation at hand.
3. The symmetry of the suggested aggregation operators concerning the parameter ensures that the ranking orders of alternatives stay generally consistent across parameter values. This stability is critical in decision-making because it prevents the outcomes from being impacted by the decision-makers' pessimism or optimism.

## 10 Conclusion and recommendation for future work

This study presents an innovative approach, the q-SFR TOPSIS method, tailored to address the challenges posed by imprecision in decision-making processes. The proposed method effectively incorporates q-SFRSs into various multi-criteria decision-making (MCDM) methodologies, extending fuzzy rough sets into a three-dimensional context. This research presents a new theory on q-spherical fuzzy rough sets together with its fundamentals involving arithmetic operations, aggregation operators, and distance measures. In the proposed fuzzy sets, distances between q-spherical fuzzy rough sets can also be Euclidean distances. The proposed q-spherical fuzzy rough TOPSIS method considers normalized Euclidean distances and calculates the closeness ratios to ideal solutions based on these distances. The performance of the SF-TOPSIS method has been compared with existing approaches. The rankings are completely the same as when the q-SFWGM operator is used. Through sensitivity analysis, we have established the reliability and robustness of the q-SFR TOPSIS method, as it consistently yields the same optimal selections regardless of variations in criteria weights. This underscores the method's stability and its ability to provide reliable recommendations across diverse decision contexts. A significant contribution of this paper lies in the introduction of a methodology tailored for scenarios involving multiple experts, addressing a gap in existing literature. By considering the input of multiple stakeholders, our approach enhances the comprehensiveness and inclusivity of decision-making processes, leading to more informed and consensus-driven decisions. The q-spherical fuzzy rough TOPSIS method has demonstrated promising capabilities in addressing decision-making problems under uncertainty. However, it's crucial to acknowledge certain limitations encountered in this study. Firstly, the method's applicability may be constrained by the complexity of decision scenarios and the availability of precise domain knowledge for defining fuzzy rough sets and determining appropriate values for the q parameter. Secondly, the computational complexity of the method may pose challenges, particularly in large-scale decision problems, necessitating further exploration of efficient algorithmic implementations and parallel computing strategies. To address these limitations and advance the field of fuzzy decision-making, several avenues for future research can be explored. Firstly, investigating novel hybridization approaches by integrating the q-spherical fuzzy rough TOPSIS method with other decision-making frameworks, such as evolutionary algorithms or machine learning techniques, can enhance its robustness and applicability across diverse domains. Secondly, exploring extensions to handle dynamic decision environments by incorporating mechanisms for adaptive weight assignment or incorporating time-varying criteria importance can improve the method's suitability for dynamic decision-making contexts.

## Funding

This project is funded by 10.13039/501100002383King Saud University, Riyadh, Saudi Arabia.

## Consent for publication

This manuscript has not been published and is not under consideration for publication elsewhere.

## Data availability statement

The accompanying manuscript does not contain any associated data. The paper only presents the written text and does not have any additional data that supports the claims and conclusions presented in the manuscript.

## CRediT authorship contribution statement

**Ahmad Bin Azim:** Conceptualization. **Asad Ali:** Conceptualization. **Abdul Samad Khan:** Data curation. **Fuad A. Awwad:** Formal analysis. **Emad A.A. Ismail:** Investigation. **Sumbal Ali:** Software.

## Declaration of competing interest

In accordance with the journal's submission guidelines, I would like to disclose any potential conflicts of interest related to this submission. I, Ahmad Bin Azim, the first author, hereby declare that I have no conflicts of interest to report in relation to the research presented in the manuscript. All authors listed have reviewed and agreed with the content of this submission.

## Appendix

FS: Fuzzyset
IFS: Intuitionisticfuzzyset
PFS: Picturefuzzyset
SFS: Sphericalfuzzysets
q−SFS: q−sphericalfuzzysets
RS: Roughset
q−SFRS: q−sphericalfuzzyroughset
IFRS: Intuitionisticfuzzyroughset
PyFRS: Pythagorean fuzzy rough set
MADM: Multi-attribute decision-making
q-ROFRS: q− rung orthopair fuzzy rough set
PFRS: Picturefuzzyroughset
q− ROFS: q− rung orthopair fuzzy set
AOs: Aggregation operators
RFID: Radio Frequency Identification
Wi-Fi: Wireless Fidelity
PyFS: Pythagorean fuzzy set
PG: Positive grade
NG: Negative grade
MCDM: Multi-criteria decision-making
AHP: Analytic hierarchy process
${q-\text{ROPFS}}_{t}S$: q− rung orthopair picture fuzzy soft set
TOPSIS: Technique for order of preference by similarity to ideal solution
q-SFRNs: q−sphericalfuzzyroughnumber
q-SFRNIS: q-spherical fuzzy rough negative ideal solution
q-SFRPIS: q-spherical fuzzy rough positive ideal solution
PIS: Positive ideal solution
NIS: Negative ideal solution
MAGDM: Multi-attribute group decision-making
